# Smart Diagnostics: Hierarchical Deep Learning of Acoustic Emission Signals for Early Crack Detection in Zirconia Dental Structures

**DOI:** 10.3390/s26092682

**Published:** 2026-04-26

**Authors:** Kuson Tuntiwong, Rangsinee Wangman, Kanchana Kanchanatawewat, Boonjira Anucul, Hiranya Sritart, Pattarapong Phasukkit, Supan Tungjitkusolmun

**Affiliations:** 1 School of Dentistry, King Mongkut’s Institute of Technology Ladkrabang, Bangkok 10520, Thailand; kuson.tu@kmitl.ac.th (K.T.); kanchana.ka@kmitl.ac.th (K.K.); boonjira.an@kmitl.ac.th (B.A.); 2 Department of Biomedical Engineering, School of Engineering, King Mongkut’s Institute of Technology Ladkrabang, Bangkok 10520, Thailand; 64601116@kmitl.ac.th (R.W.); supan.tu@kmitl.ac.th (S.T.); 3 Department of Medical Technology, Faculty of Allied Health Sciences, Thammasat University, Pathumthani 12120, Thailand; hiranya.s@allied.tu.ac.th

**Keywords:** acoustic emission, crack detection, deep learning, dental zirconia, gated recurrent units (GRU), long short-term memory (LSTM), nondestructive testing, pencil lead  break (PLB) test, scalogram

## Abstract

Monolithic zirconia restorations are frequently affected by the unnoticed growth of subcritical cracks, a failure process that is not captured by traditional imaging methods like radiographs and ultrasounds in sophisticated dental architectures. To address this evaluative inadequacy, this research introduces a hierarchical deep learning framework for microcrack detection and spatial localization. We promote a hierarchical deep learning system that integrates Acoustic Emission (AE) detection alongside signal processing. Raw AE signals utilized during dynamic loading are enhanced via Kalman filtering and Continuous Wavelet Transform (CWT) to construct high-fidelity time–frequency scalograms. The diagnostic pipeline operates in two stages: first, a hybrid CNN–BiGRU network with temporal attention fulfills zirconia component-level classification; second, a ResNet-18 backbone integrated with Bidirectional LSTM and Multi-Head Attention precisely localizes defects across five anatomical crown regions. This hierarchical design effectively captures the non-stationary, transient nature of fracture-induced stress waves. The framework achieved an F1-score of 99.00% and an AUC of 0.994, significantly outperforming conventional convolutional networks. By enabling predictive maintenance through early, non-invasive damage localization, this study demonstrates a promising laboratory framework for AE-based crack detection in zirconia dental structures and prosthetics and toward enhanced clinical reliability in digital dentistry.

## 1. Introduction

Zirconia ceramics have significantly transformed the field of dentistry and are rapidly emerging as the material of choice for intricate dental applications [1]. Excellent mechanical strength, exceptional biocompatibility, and cosmetic fidelity that closely resemble natural dentition are the unique features that led to this decision [2]. The common use of yttria-stabilized tetragonal zirconia polycrystals (Y-TZPs) in creating dental crowns, fixed partial dentures, implant abutments, and fixtures [3] shows their strong ability to handle significant, repeated biting forces. These biting forces can range from 200 to 900 N in oral cavities [4]. They also keep their shape and function well in a harsh oral environment. Clinical studies have highlighted this success, with survival rates for Y-TZP-based restorations consistently exceeding 95% over a five-year observation period [5]. Zirconia is nevertheless vulnerable to low-temperature degradation (LTD), also known as temporal degradation and subcritical crack propagation, despite these convincing clinical findings [6]. Combining chronic anomalous chewing function with stress-corrosion fracture processes, such as bruxism and clenching, can lead to catastrophic component breakage [7]. The primary failure mechanisms that impact both structural integrity and clinical longevity are the presence of intrinsic flaws and the subsequent initiation and propagation of microcracks [8]. Crack propagation is in accordance with the principles of fracture mechanics. In Zirconia, the increase in fracture toughness increases exponentially as the applied stress intensity factors increase, especially when the material is subjected to the corrosive aqueous conditions present in the oral cavity [9]. Therefore, the development of sensitive, non-invasive methods for the early identification of microdamage is essential to the transition in dentistry to a trustworthy, predictive maintenance paradigm.

### 1.1. Limitations of Conventional and Current Nondestructive Evaluation (NDE)

Standard techniques for evaluating the mechanical strength of ceramics include Vickers indentation, fracture toughness testing (e.g., the Single-edge V-notch beam), and traditional methods like ISO 6872:2024 flexural tests [10]. These methods provide useful information. However, their destructive nature limits their use in clinical settings [11]. The indentation fracture (IF) method is often preferred because it is simpler to use. Still, to assess the long-term safety of high-strength zirconia, we need more sensitive, noninvasive diagnostics. This restricts their use for real-time monitoring of dental structures. Evaluating the structural integrity of these dental materials requires effective methods to detect damage early. Dental research has explored alternative NDE techniques, including ultrasonic pulse-echo and radiographic imaging [12,13]. Identifying microstructural changes and microcracks smaller than 100 µm is difficult [14]. This challenge arises mainly because these methods often lack the necessary sensitivity. There are practical limitations as well. Due to the complex shapes of dental crowns or implant components, traditional ultrasonic testing is less effective. Traditional radiography only achieves 9.1–11.3% sensitivity for detecting cracks because it requires precise alignment of the X-ray beam with the crack. Although more recent techniques, such as cone-beam computed tomography (CBCT), increase detection rates, they frequently fail to detect cracks smaller than 0.2 mm. Radiation and artifacts from high-density zirconia are additional issues with these techniques. Although micro-CT can resolve features down to 5–20 µm, it requires radiation doses of 100–150 kVp. This exceeds the clinical limits of 60–90 kVp. Although it requires direct contact and performs poorly on intricate three-dimensional shapes, ultrasound microscopy can differentiate materials based on their acoustic impedance. Visual inspection exhibits only 23–35% sensitivity. To address this, we suggest a new framework that combines AE sensors with Deep Learning. This approach allows for the detection of small microcracks in zirconia dental materials. It provides a more effective diagnostic tool for both restorations and implant components.

### 1.2. Acoustic Emission and the Combination with Deep Learning for Damage Detection

AE monitoring is a novel approach to structural health monitoring (SHM) [15,16]. AE has shown itself to be a successful nondestructive assessment technique for monitoring developing defects. Without requiring external energy, AE passively detects transient elastic waves released during material deformation [17]. To minimize signal loss and reduce background noise, it operates between 100 and 700 kHz. For further exploration of the AE source, some investigations associated AE signals with microstructures or micro-damage events in the material. The real-time detection and analysis of transient elastic waves produced by the rapid release of stored strain energy during active material degradation processes. AE provides important benefits with its real-time capabilities and high sensitivity to tiny damage events before they become noticeable failures [18,19]. This is different from passive inspection methods. Piezoelectric sensors, which retain important information about the damage mechanism, location, and severity in their temporal and spectral properties, are used to convert these waves into unique waveforms [20,21]. Pan et al. [22] further demonstrated that GMM clustering of AE parameters can successfully distinguish multiple damage modes—including matrix cracking, debonding, and fiber tearing—based on their distinct frequency and energy signatures. Although AE technology is widely used in SHM in the aerospace, civil, and mechanical engineering domains [23,24], its application in dental materials remains relatively limited. Recent studies have demonstrated the feasibility of automated crack detection in monolithic zirconia crowns using AE signals combined with deep learning techniques [25]. Currently, the development of Artificial Intelligence (AI) is rapidly closing this knowledge gap, particularly with the advent of sophisticated deep learning architectures. When creating hierarchical feature representations from spatial or spatiotemporal data, Convolutional Neural Networks (CNNs) perform better than Recurrent Neural Networks (RNNs), especially Gated Recurrent Units (GRU) and Long Short-Term Memory (LSTM) [26,27,28]. The complex temporal relationships present in sequential sensor data are best modeled by them. Internal memory states that capture long-range dependencies are stored in LSTM and GRU. GRU uses only reset and update gates to simplify LSTM. It provides comparable performance at reduced computational costs. Liao et al. showed that CNN-LSTM achieved 92% higher than CNN and RNN architectures [29]. Nasrullah and Zhao studied how to classify musicians using a temporal architecture. A GRU model is used to capture temporal characteristics. The overall F1-score was 93.7% across three independent tests [30]. Both temporal progressions and instantaneous signatures were successfully captured. Transient, nonstationary AE signals are not well handled by classical Fourier analysis. The Short-Time Fourier Transform (STFT) imposed fixed time–frequency resolution trade-offs. The CWT provides an adaptive multiresolution analysis. Additionally, nonstationary AE waveforms can be decomposed into multiresolution scalograms using sophisticated signal processing techniques, such as the CWT. These unique two-dimensional representations are highly discriminative input features for deep learning classification [31]. Zhou et al. [32] proposed a deep residual network (DRN) employing CWT scalogram images for zone-based AE source localization in a steel-concrete composite slab, achieving accuracy exceeding 99% at 3–4.5 cm resolution under multiple damage states. Although AI-driven NDE has shown success in industrial applications, achieving classification accuracy exceeding 90% for defect characterization [33], a full investigation combining real-time AE monitoring, AI-driven signal classification, and microstructural analysis that is particularly appropriate for complex dental ceramic systems is obviously a bit beyond academic literature.

### 1.3. Current Study and Translational Impact

The current study is an application-driven contribution that directly addresses this unresolved clinical need. The novelty lies in three aspects: (i) AE-based SHM applied to dental zirconia, a domain with distinct geometric and localization challenges; (ii) a hierarchical two-stage architecture reflecting the clinical examination workflow; and (iii) a domain-adapted acoustic transmission rod that conducts AE waves through direct contact with the crown surface, reducing physiological interference from saliva, soft-tissue coupling, and oral biofilm. We use a servo-hydraulic universal testing machine (UTM) to subject anatomically accurate zirconia crowns to control quasi-static and cyclic fracture loading while simultaneously obtaining high-fidelity AE data from strategically placed piezoelectric sensors in an experimental study. Scalograms are created by carefully preprocessing the raw AE waveforms using CWT. These scalograms are used to train a hybrid CNN-GRU and a ResNet-18 architecture integrated with Bidirectional Long Short-Term Memory (BiLSTM) and Multi-Head Attention models, as well as other deep learning architectures, to accomplish autonomous component geometry classification and accurately localize crack initiation and propagation events. Classification performance is carefully validated using established metrics. Prior to experimental loading, AE signals were first collected using PLB tests according to ASTM guidelines to validate signal integrity and calibrate sensor sensitivity [34]. To enable predictive maintenance procedures in clinical dentistry, this integrated approach demonstrates how AE-based diagnostics, as a hierarchical deep learning approach, can be utilized to detect cracks in zirconia materials early and accurately. This research aims to create and confirm a combined diagnostic framework. This framework uses hierarchical deep learning and AE-based monitoring to find cracks in zirconia dental restorations and implant-like structures. It focuses on early and precise detection in conditions like those found in clinical settings. This approach will help to improve patient safety. In addition to the functional analysis, high-resolution Scanning Electron Microscopy (SEM), which utilizes secondary and backscattered electron imaging of fracture surfaces. To provide condition-based maintenance, AI-driven predictive analytics, and real-time AE monitoring. The findings have significant translational implications for developing tailored dental healthcare delivery systems, improving clinical decision-making, and reducing the risk of iatrogenic outcomes.

This portion of the paper is organized as follows: Section 1 covers the research background and a review of relevant literature on (i) zirconia and the cause of material failure, (ii) limitations of IF testing and current NDE, (iii) AE combined with deep learning for defect detection, and (iv) the current methods. Section 2 establishes the experimental framework, detailing the fabrication of an anatomically accurate zirconia sample, the data-acquisition protocols, and the Kalman-filtering-based signal-conditioning pipeline. This section further elucidates the proposed hierarchical deep learning architecture, integrating CNN–BiGRU and ResNet–18 with Multi-Head Attention mechanisms. Section 3 describes system validation and on-site experimental setup. In Section 4, we present the results of the developed algorithm using field data from the UTM. Section 5 provides a discussion of the research, outlining its limitations and future research possibilities. Finally, Section 6 concludes the study, summarizing the contributions of this intelligent diagnostic framework to digital dentistry.

## 2. Materials and Methods

### 2.1. Zirconia Sample Fabrication

#### Zirconia Crown and Dental Implant Shape-like Sample Preparation

The following section describes the methods used for sample preparation, material property evaluation, fabrication techniques, and data collection systems employed during this research. Zirconia samples with implant-shaped and full-contour dental crown designs were used to study AE behavior when cracks initially form and propagate through the material under laboratory-controlled conditions. The implant shape-like sample was designed as a cylindrical shape using a zirconia disc (Shofu Disc ZR Lucent; SHOFU, Kyoto, Japan) and fabricated with a computer-aided design/computer-aided manufacturing (CAD/CAM) workflow. The key material specifications of the zirconia disc are summarized in Table 1.

The zirconia sample was fabricated using a five-axis CAD/CAM milling system (inLab MC X5, Dentsply Sirona, Bensheim, Germany), capable of processing zirconia discs (Ø98.5 mm) with high-speed spindle operation (up to 60,000 rpm). Digital design files were processed using inLab CAM software (inLab; Version 22.8, Dentsply Sirona, Bensheim, Germany) within an open STL architecture workflow.

For the crown sample, a plastic artificial tooth (A55AN-111; Nissin Dental Products Inc., Kyoto, Japan) was digitally scanned to generate a master reference model for zirconia crown fabrication. The sample underwent standard full-coverage crown preparation and was digitized using an intraoral scanner (CEREC Primescan AC v5.2.13; Dentsply Sirona, Bensheim, Germany). The crowns were fabricated from the same zirconia material under identical milling conditions, followed by sintering (inLab Profire; Dentsply Sirona, Bensheim, Germany) at temperatures up to 1650 °C with controlled heating rates (≤120 °C·min^−1^). Follow-up glazing and porcelain firing were performed using a dental ceramic furnace (Multimat Cube Press; DeguDent GmbH–Dentsply Sirona, Hanau-Wolfgang, Germany), operating at temperatures up to 1200 °C with programmable multi-stage heating and controlled pressing conditions.

### 2.2. Experimental Setup and Data Acquisition

#### 2.2.1. Acoustic Emission (AE) Measurement

To enable high-sensitivity detection of transient elastic waves generated during microcrack initiation and propagation, this study used a wideband AE sensing system. The WM500/1 (QingCheng AE Institute Co., Ltd., Guangzhou, China) functions as a small passive piezoelectric AE sensor that provides a broad frequency output across its operational range. The sensor operates between 100 kHz and 1000 kHz, with a sensitivity peak exceeding 65 dB, allowing it to detect brief AE signals that occur when brittle materials develop microcracks. The AE sensor was used with a petroleum jelly couplant connected to the zirconia sample to ensure effective acoustic signal transfer while reducing signal strength. The technical specifications of the AE sensor used in this study are summarized in Table 2.

AE signals were collected using a two-channel, 16-bit digital oscilloscope (Picoscope 4262; Pico Technology, St Neots, UK) with a sampling rate of 2 MS/s. The technical specifications of the data acquisition system are summarized in Table 3. The AE monitoring system was employed to capture real-time acoustic responses during the mechanical loading of zirconia crowns and implant shape-like samples. The collected AE signals were then processed and used to train the model.

#### 2.2.2. Testing Measurement

The UTM (Shimadzu AG-100kNX2; Shimadzu Corporation, Kyoto, Japan) was calibrated before the testing session in accordance with the manufacturer’s recommendations. The controlled loading testing system includes its fundamental technical details, which are presented in Table 4. A round stainless steel bur with a long shank (diameter: 1.4 mm) was attached to the load cell through a fixture to apply localized compressive loading to the zirconia crown sample. The crosshead speed was maintained at a constant rate of 1 mm/s at an angle of 135° from the long axis of the tooth during testing. Fracture locations were identified under SEM.

Before conducting mechanical loading experiments, the AE system was calibrated, and signals were validated using PLB tests following ASTM E976 [34,35,36]. A 0.5 mm diameter, 2H pencil lead with a break length of 2–3 mm was fractured at an angle of approximately 30° relative to the sample surface. PLB events were performed on the right, left, anterior (labial), posterior (palatal), and apical (incisal) surfaces of both the cylinder-like implant fixture (root) and crown sample. A total of 300 PLB events were performed on each surface to ensure signal reproducibility under consistent acquisition conditions.

### 2.3. Signal Preprocessing Pipeline

After completing mechanical loading and AE data acquisition under controlled experimental conditions, the recorded AE waveforms were subjected to systematic signal preprocessing and time–frequency transformation prior to feature extraction and deep learning-based analysis. These steps aim to reduce noise, normalize amplitudes, and maintain transient crack-related features.

#### 2.3.1. Kalman Filter for AE Signal Denoising

The AE signals detected during mechanical loading show random variations caused by ambient noise, sensor connection differences, and electronic interference. To reduce these effects while maintaining transient crack-related signal components, a Kalman filter was used as a state estimation-based denoising method, which has been widely employed for stochastic signal estimation and damage-related AE analysis [37,38].

The AE signal was modeled using a discrete-time linear state-space representation, as given in Equations (1) and (2).

(1) $$x_{k}=Ax_{k-1}+w_{k}$$

(2) $$z_{k}=Hx_{k}+v_{k}$$

where $x_{k}$ denotes the hidden true signal state and $z_{k}$ represents the measured waveform. The process noise $w_{k}$ and measurement noise $v_{k}$ are assumed to be zero-mean Gaussian variables with covariance matrices Q and R, respectively.

**Figure 1:**
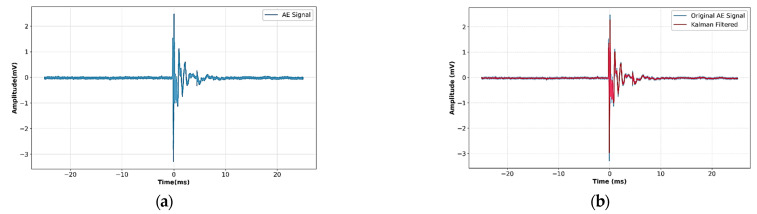
AE signal preprocessing: (**a**) Raw AE signal before denoising. (**b**) AE signal after Kalman filtering.

The recursive prediction–update mechanism estimates the optimal state by minimizing the mean squared estimation error through the Kalman gain formulation. This adaptive filtering effectively reduces baseline noise while maintaining high-frequency burst components associated with microcrack initiation. Representative denoising results are shown in Figure 1. Figure 1a displays the raw AE signal before preprocessing, while Figure 1b shows the filtered signals with decreased baseline fluctuations and preserved high-frequency burst components related to crack events.

#### 2.3.2. Z-Score Normalization

After denoising, Z-score normalization was applied to standardize the amplitude distribution of AE signals before extracting features and performing analysis with deep learning methods. This normalization minimizes amplitude bias caused by fluctuations in sensor coupling, signal attenuation, and sample geometry, and it has been widely used in signal preprocessing for machine learning-based damage detection [39,40]. The Z-score normalized signal x prime is defined as in Equation (3).

(3) $$x^{\prime }\left(t\right)=\frac{x\left(t\right)-\mu }{\sigma }$$

where x(t) is the denoised AE signal, μ is the mean value of the signal, and σ is the corresponding standard deviation.

This normalization ensures that all signals are on a similar scale with zero mean and unit variance, thereby improving numerical stability and improving convergence during deep learning model training.

#### 2.3.3. Continuous Wavelet Transform and Scalogram Construction

Due to the non-stationary and transient nature of AE signals, time–frequency analysis is required to capture both temporal localization and frequency-dependent characteristics of crack-related events. This study used the CWT to generate time–frequency representations of AE signals through scalograms, which serve as standard tools for analyzing transient signals and detecting damage via AE. The CWT of a signal is defined as Equation (4).

(4) $$CWT\left(a,b\right)=\frac{1}{\sqrt{\left|a\right|}}\int_{-\infty }^{\infty }x(t)\psi^{*}\left(\frac{t-b}{a}\right)dt$$

where a is the scale parameter related to frequency, b is the time-shift parameter, ψ(t) is the mother wavelet, and ${\left(\cdot \right)}^{*}$ denotes complex conjugation.

The magnitude of the CWT coefficients creates a scalogram, offering a two-dimensional view of signal energy distribution across time and frequency. The scalograms clearly show high-frequency burst components corresponding to crack initiation events, along with lower-frequency structural responses. These image-like representations serve as input features for the following CNN architectures.

**Figure 2:**
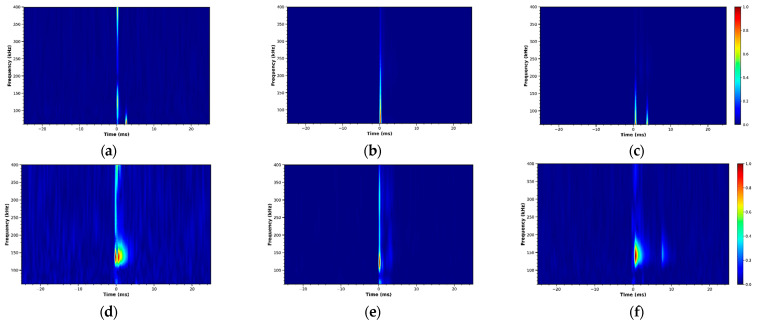
CWT scalograms of AE signals from different sample components and crown regions: (**a**) root component; (**b**) incisal; (**c**) labial; (**d**) left; (**e**) palatal; (**f**) right.

Representative scalograms from the zirconia sample are shown in Figure 2, including signals from the root component and five anatomical regions of the crown (Incisal, Labial, Left, Palatal, and Right). Distinct time–frequency energy patterns can be observed among these regions, reflecting variations in stress-wave propagation and crack-related acoustic signatures. These distinct patterns provide informative features that allow the hierarchical deep learning framework to perform both component classification and spatial crack localization.

### 2.4. Hierarchical Deep Learning Framework

**Figure 3:**
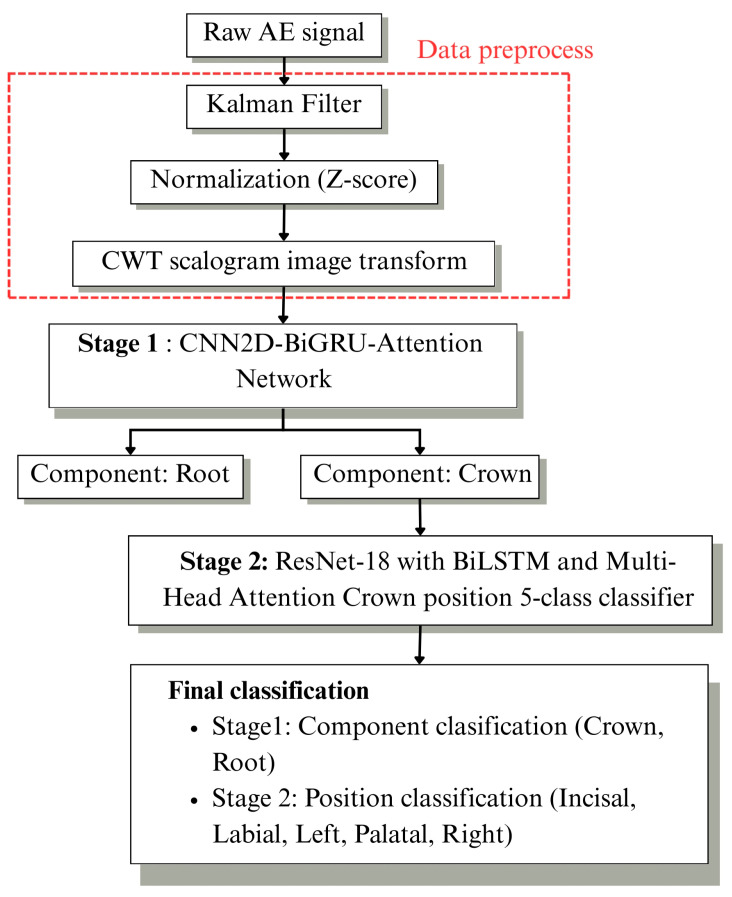
Proposed hierarchical deep learning framework for AE-based crack diagnosis.

After the time–frequency transformation, a hierarchical deep learning framework was developed for crack diagnosis, as illustrated in Figure 3. The proposed system consists of two sequential stages designed for component-level classification and detailed crack localization.

#### 2.4.1. Stage1: Component Discrimination via CNN–BiGRU

The CNN–BiGRU architecture with temporal attention was developed to classify crack classifications into root and crown regions of the zirconia sample. The hybrid system employs CNNs to extract spatial features from scalogram images and bidirectional gated recurrent units to analyze complete temporal sequences before using attention to identify the most important temporal features for binary classification.

The convolutional stage uses 2D convolution layers with ReLU activation and max pooling to extract spatial features from scalogram images. The convolution operation is described by Equation (5).

(5) $$y^{l}=\sigma (W^{l}*x^{\left(l-1\right)}+b^{l})$$

where $y^{l}$ is the output feature map at layer l, $W^{l}$ is a learnable weight kernel (convolutional filter), $x^{\left(l-1\right)}$ is the input feature map from the previous layer, $b^{l}$ is a bias term, σ is the ReLU activation function and ∗ is a convolution operation.

The extracted features pass through a bidirectional GRU layer that processes sequences in both directions. The GRU hidden state is calculated as in Equation (6).

(6) $$h_{t}=\left(1-z_{t}\right)⊙h_{\left(t-1\right)}+z_{t}⊙tanh(W_{h}x_{t}+U_{h}\left(r_{t}⊙h_{\left(t-1\right)}\right)+b_{h})$$

where $h_{t}$ is the hidden state at time t, $z_{t}$ is update gate, $r_{t}$ is a reset gate, $x_{t}$ is the input vector, $W_{h}$ and $U_{h}$ are weight matrices, $b_{h}$ is a bias vector and ⊙ is element-wise multiplication.

The temporal attention layer projects BiGRU output from 256 to 128 dimensions, calculates attention scores, and normalizes them using softmax across 196-time steps. Temporal attention is calculated as Equation (7).

(7) $$\alpha_{t}=\frac{exp(e_{t})}{\sum_{\tau =1}^{196}exp(e_{\tau })}$$

The weighted sum $c=\sum_{\tau =1}^{196}\alpha_{t}h_{t}$ produces the attention output vector (*B*, 256).

The attention-weighted output is passed through fully connected layers with dropout, and the final layer uses softmax activation for binary classification, as shown in Equation (8).

(8) $${\hat{y}}_{i}=\frac{exp(z_{i})}{\sum_{j=1}^{2}exp(z_{j})}$$

The network is trained using categorical cross-entropy loss. Based on the argmax output, if argmax = 1 (root), the analysis terminates; if argmax = 0 (crown), the sample is forwarded to the subsequent processing stage for detailed crack area identification.

#### 2.4.2. Stage2: Spatial Crack Localization via ResNet-18 with LSTM Architecture and Multi-Head Attention

The proposed Stage 2 architecture performs detailed crack localization within zirconia crown regions using five-class classification: incisal, labial, left, palatal, and right. It employs ResNet-18 as the deep residual feature extraction backbone for the scalogram input. Four residual stages, with the spatial down sampling factor increasing by one at each stage, are used to extract hierarchical spatial–frequency features. The spatial dropout is applied before vectorizing the feature map for contextual feature learning.

A two-layer BiLSTM network processes sequential features in both forward and backward directions. To improve localization sensitivity, a Multi-Head Attention mechanism is used to highlight informative crack-related regions within the sequential features. The attention operation is defined as Equation (9).

(9) $$Attention\left(Q,K,V\right)=softmax\left(\frac{QK^{T}}{\sqrt{d_{k}}}\right)V$$

where *Q*, *K*, *V* are query, key and value matrices from BiLSTM output, and $d_{k}$ is the key dimension. The output is passed through layer normalization and dropout.

#### 2.4.3. Model Training Configuration and Optimization

To ensure reproducibility and unbiased evaluation, consistent dataset partitioning and optimization strategies were adopted for both stages. For Stage 1, which performs component classification between Crown and Root categories, a total of 3000 AE signal samples were used, comprising 1500 signals per class. The dataset received an 80%/20% train–test split, producing 2400 training samples and 600 test samples, while maintaining equal class distribution. Stage 2 conducts crown position classification for five anatomical areas, including Incisal, Labial, Left, Palatal, and Right. A total of 1500 AE signal samples were used, with 300 samples assigned to each class. The same 80%/20% train–test split was applied, yielding 1200 training samples and 300 test samples. The hierarchical routing strategy required Stage 2 training to focus on Crown-related samples selected during dataset preparation.

The PLB acquisition protocol in this study follows a Spatial Characterization approach, in which 300 PLB signals per surface position are acquired from a single dedicated sample to map the position-specific acoustic waveform signatures characteristic of each anatomical location within that crown geometry (Baseline mapping). This protocol enables the deep learning model to learn the acoustic waveform differences associated with each anatomical position, grounded in the wave propagation physics of the crown structure rather than in sample-to-sample morphological variation. For Stage 1 (Root vs. Crown), one root and one crown sample were used; all PLB signals were acquired from these samples, and an 80%/20% signal-level split was applied, yielding 2400 training and 600 test signals. For Stage 2 (five-class crown position), one crown sample was used; all 1500 PLB signals (300 per class across five positions) were acquired from this sample, and an 80%/20% signal-level split yielded 1200 training and 300 test signals. Each signal comprises 100,000 data points sampled at 2 MS/s. Within this Spatial Characterization framework, signal-level splitting provides a valid evaluation of whether the model learns class-discriminative spectral features from the crown geometry.

Model parameters were optimized using the AdamW optimizer with early stopping to prevent overfitting. A learning rate scheduling strategy was also applied in Stage 2 to improve convergence stability. Detailed hyperparameter configurations for both stages are summarized in Table 5.

The model’s performance evaluation included accuracy measurements, along with precision, recall values and F1-score calculations, and confusion matrix analysis to determine the overall classification success rate. The accuracy indicated the model’s ability to correctly identify all samples, while precision and recall assessed its effectiveness in detecting positive predictions and recognizing true class instances. The F1-score combined precision and recall values to calculate their harmonic mean, balancing sensitivity and specificity in multi-class classification systems. The confusion matrix analysis examined how the model performed across specific classes to identify patterns that could lead to incorrect predictions between different anatomical regions and component types. The evaluation metrics were applied uniformly in both Stage 1 and Stage 2, allowing for an equal assessment of performance throughout the hierarchical system.

## 3. System Validation and On-Site Mechanical Testing

### 3.1. Sample Preparation and AE Sensor Installation

The experimental samples were produced as described in the Section “Zirconia Crown and Dental Implant Shape-like Sample Preparation”.

**Figure 4:**
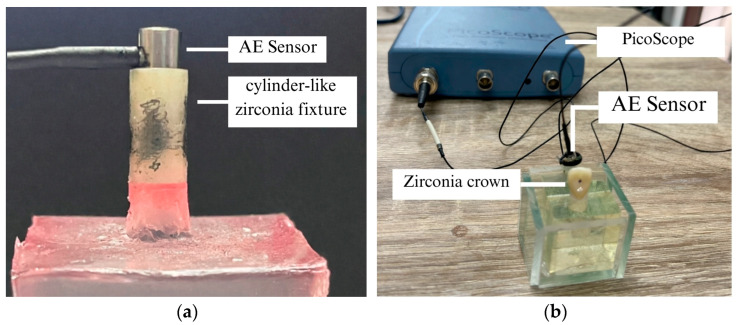
Experimental setup of zirconia sample: (**a**) implant-like cylindrical fixture with top-mounted AE sensor; (**b**) zirconia crown with labial-mounted AE sensor and data acquisition system.

From this zirconia blank, a hollow cylindrical zirconia fixture was fabricated to function as a simplified substitute for a dental implant root. As shown in Figure 4a, the fixture was vertically embedded in a rigid supporting base to ensure mechanical stability during testing. The sample was designed as a hollow cylinder with a closed bottom, featuring a concentric internal cavity that was machined to create a uniform wall thickness of 0.5 mm while preserving a solid base. This design allowed for controlled evaluation of crack initiation and AE behavior under axial load, while reducing geometric complexity associated with threaded implant structures. The sensor was mounted on the top surface of the cylindrical zirconia fixture to detect stress-wave signals that appeared when cracks first formed. A coupling agent was applied to facilitate smooth wave transmission between the zirconia surface and the sensor.

In addition, a cylindrical zirconia fixture and a zirconia crown sample were used for crack localization analysis. The crown surface was systematically divided into five anatomical regions: incisal, labial, left, palatal, and right. The Stage 2 deep learning framework used these predefined regions as classification labels to achieve structured spatial crack localization. The sensor was mounted in contact with the labial surface of the zirconia crown sample, as illustrated in Figure 4b. A metallic transmission rod was used to transmit stress-wave signals generated within the crown sample to the AE sensor. The rod was mechanically coupled to the crown at the designated loading region and aligned to facilitate efficient wave propagation toward the sensor. This direct- contact rod configuration was adopted with the clinical intraoral environment: by conducting AE stress waves through a solid, mechanically coupled pathway, the design reduces the influence of physiological factors—including saliva films, soft-tissue acoustic coupling, and oral biofilm—on the recorded waveform, compared with surface-mount sensor arrangements that are exposed to the full oral medium. The system required precise stabilization to prevent signal degradation caused by tiny openings or incorrect placement of equipment.

### 3.2. PLB-Based Signal Acquisition

**Figure 5:**
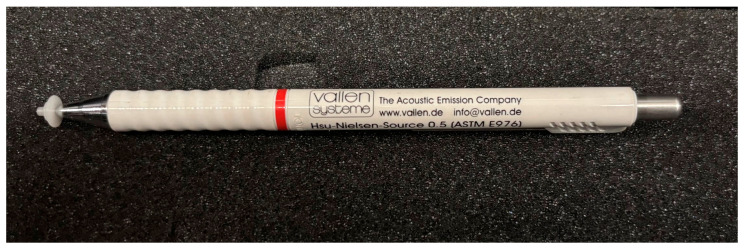
PLB source used for AE signal generation in accordance with ASTM E976.

PLB tests were conducted following ASTM E976 to create controlled and repeatable signals for dataset development. The PLB excitation source used in this study is shown in Figure 5. A 0.5 mm diameter 2H pencil lead was fractured at a length of 2–3 mm and at an angle of approximately 30° relative to the sample surface to generate consistent transient crack-like stress waves.

Prior to dataset collection, the AE sensor sensitivity was validated by performing amplitude testing at each PLB surface position. For the Crown sample, average peak amplitudes ranged from approximately 12.3 dB (Left position) to 35.8 dB (Palatal position). For the Root sample, average peak amplitudes ranged from approximately 30.8 dB (Anterior position) to 39.8 dB (Apex position). These amplitude levels, recorded consistently across all five trials per position, confirm that the AE sensor captures reproducible signals across all anatomical positions under the applied acquisition conditions.

**Figure 6:**
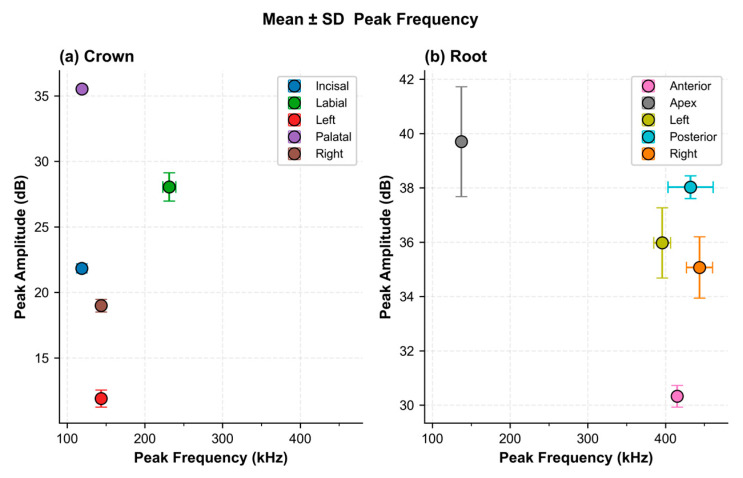
Mean ± SD of peak AE frequency (kHz) and peak amplitude (dB) per PLB excitation position for (**a**) Crown sample and (**b**) Root sample.

Figure 6 shows the mean ± SD of peak AE frequency and amplitude per PLB position. Low within-position SD confirms reproducible signal acquisition at each anatomical label. Positions are well-separated in the frequency–amplitude space: for the Crown, the Palatal position yields the highest amplitude (~36 dB, ~130 kHz) and the Left the lowest (~12 dB); for the Root, the Apex yields (~40 dB at ~130 kHz) while remaining positions cluster at 380–450 kHz. Between-position separation is consistent with path-length and wave reflection differences, providing the physical basis for position-specific discriminative features in the CWT scalograms.

**Figure 7:**
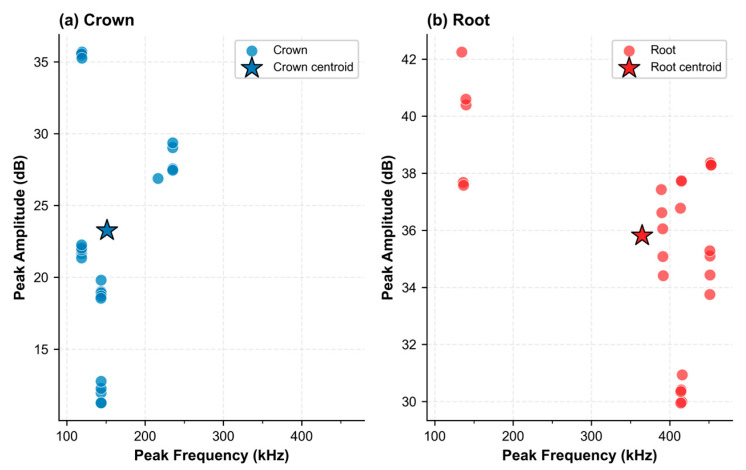
Individual PLB measurements of peak AE frequency (kHz) and peak amplitude (dB): (**a**) Crown and (**b**) Root. Stars denote the overall centroid of each dataset.

Figure 7 shows all individual PLB data points (*n* = 25 per sample type) with the dataset centroid. Crown data form two clusters: low-frequency (~110–145 kHz, 12–36 dB) and mid-frequency (~230–260 kHz, 26–29 dB), with a centroid at ~145 kHz/23 dB. Root data cluster at low frequency (~130–145 kHz, 37–42 dB) and mid-to-high frequency (~380–450 kHz, 30–38 dB), with centroid at ~360 kHz/35.5 dB. Within-cluster scatter reflects natural PLB trial-to-trial variability; between-cluster separation reflects position-specific propagation paths. Together, Figure 6 and Figure 7 confirm within-position reproducibility and between-position discriminability, both of which underpin the classification performance of the proposed framework. During signal acquisition, the AE sensor was positioned on the sample interface using a coupling agent to ensure effective stress-wave transmission and reduce attenuation. All PLB tests were performed under the same acquisition settings to ensure consistent signals. In this study, the hollow cylindrical zirconia fixture was identified as the root component, while the zirconia crown sample was identified as the crown component. A total of 3000 AE signals were collected through controlled PLB excitation. The dataset included 1500 signals from the root component, a hollow cylindrical zirconia fixture, and 1500 signals from the crown component, a zirconia crown sample. This balanced dataset was used for Stage 1 component classification.

For Stage 2 crown position classification, the 1500 crown signals obtained from PLB testing were used, evenly divided into five categories of 300 samples each. PLB excitation locations on the zirconia crown were defined and labeled by dentists using standard dental anatomical landmarks consistent with clinical tooth morphology. The five regions—Incisal, Labial, Left, Palatal, and Right—correspond directly to established clinical surface designations used in dental examination. Each PLB event was applied at a fixed anatomical location, and the resulting AE signal was assigned to the corresponding region label, ensuring that spatial labels carry direct clinical meaning and are reproducible across samples.

For Stage 1 component classification, the spatial labeling of the hollow cylindrical root fixture differs from the crown owing to its distinct geometry. Unlike the crown, the root fixture lacks anatomical surface landmarks; spatial labeling was therefore defined according to the fixed specimen orientation during signal acquisition. The fixture was firmly established in a standardized orientation relative to the sensor throughout all PLB tests, and region labels were allocated based on the geometric location of the PLB excitation point in relation to this fixed reference framework. This orientation-based labeling ensures consistent source–sensor relationships and reliable interpretation of signal attenuation and guided-wave propagation in the axisymmetric structure.

### 3.3. Stage 1: CNN2D–BiGRU Component Classification

The PLB-based dataset was used to develop and assess stage 1 of the proposed hierarchical diagnostic framework. This stage focuses on binary classification of components between root and crown signals before detailed crack localization.

**Figure 8:**
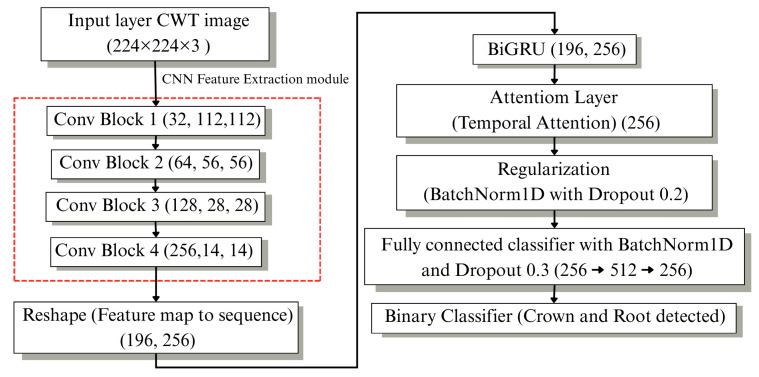
CNN–BiGRU architecture for Stage 1 component classification.

CWT-based scalogram images are initially processed by a CNN feature extraction module to learn spatial features. These feature maps are then reshaped into sequences and fed into a bidirectional GRU (BiGRU) to capture temporal dependencies. A temporal attention mechanism is subsequently applied to emphasize informative crack-related segments. The attention-weighted features are regularized and passed through a fully connected layer with softmax activation for binary classification between root and crown categories, as shown in Figure 8.

Detailed architectural configurations and training hyperparameters are summarized in Table 6. The model was trained using cross-entropy loss and optimized with AdamW on the balanced PLB dataset (3000 signals; 1500 root and 1500 crown samples) with an 80%/20% train–test split. The results demonstrate strong discrimination capability between component types, confirming the effectiveness of the CNN–BiGRU architecture as the initial classification stage within the hierarchical crack diagnostic framework.

### 3.4. Stage 2: ResNet-18 Integrated with BiLSTM and Multi-Head Attention Crown Position Classification

**Figure 9:**
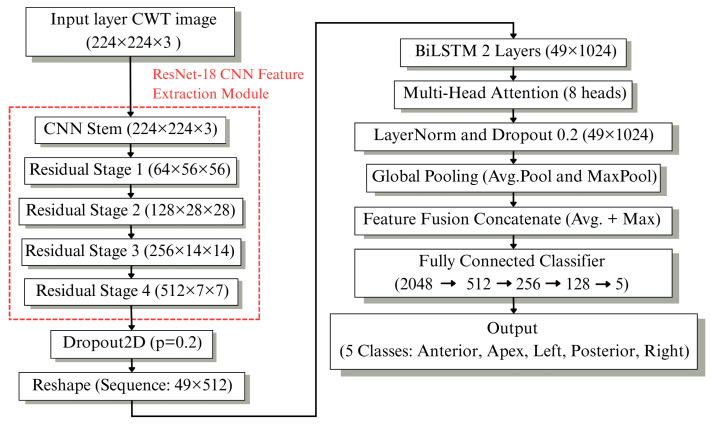
Architecture of the ResNet-18–BiLSTM model with multi-head attention for crown position classification.

The second stage of the proposed hierarchical framework was designed for spatial crack localization within the zirconia crown. The model receives CWT-based scalogram images resized to 224 × 224 × 3 as input. As shown in Figure 9, the architecture includes a ResNet-18 feature extraction backbone, followed by bidirectional sequential modeling and attention-based feature refinement.

A ResNet-18 backbone is used to extract hierarchical spatial features from the input CWT scalograms. The input images are processed through a CNN stem and four residual stages with increasing spatial down sampling. Spatial Dropout2D is applied for regularization before reshaping the feature maps into a sequential format for contextual modeling. A two-layer Bidirectional LSTM processes the sequence features, followed by a Multi-Head Attention module that enhances the representation by emphasizing informative spatial–frequency regions. Layer normalization and dropout are used to stabilize the training process.

The attention-enhanced features are aggregated globally using both average and max pooling. The pooled representations are then combined through concatenation. After several fully connected layers, the final softmax output layer provides the probability scores for the five crown regions: incisal, labial, left, palatal, and right. Detailed training and architectural hyperparameters are summarized in Table 7.

### 3.5. Mechanical Loading-Based Onsite Validation

**Figure 10:**
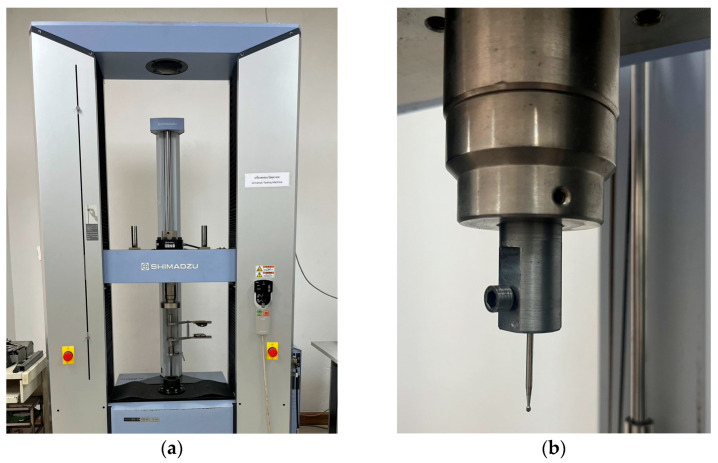
Mechanical loading setup for crown crack localization: (**a**) UTM; (**b**) close-up of the loading tip applied to the zirconia crown.

To validate the proposed framework within clinically relevant conditions, an onsite validation dataset (*n* = 30) was established through controlled mechanical loading conducted on a zirconia crown sample. Utilizing a UTM (Shimadzu, Japan), mechanical loading was performed, as shown in Figure 10a. A spherical-tip metallic indenter was mounted on the crosshead of the testing machine to ensure controlled and localized contact loading, as illustrated in Figure 10b.

**Figure 11:**
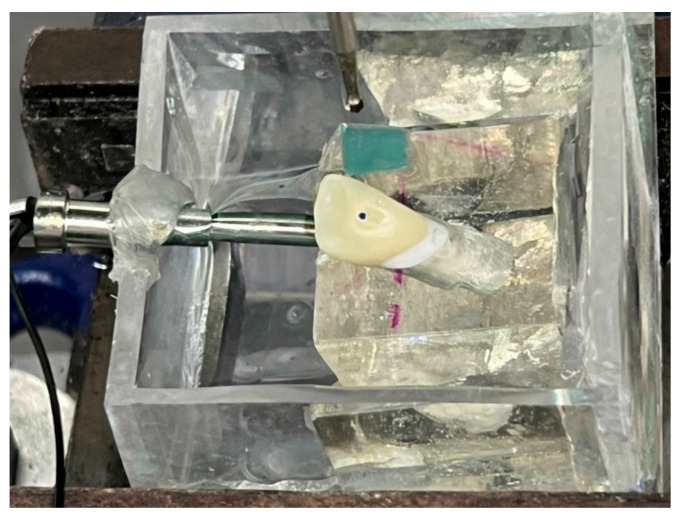
Zirconia crown sample mounted at a 135° inclination in a customized fixture.

To simulate physiological chewing conditions, the zirconia crown sample was positioned at an inclination of 135° relative to the force application axis. This angle reflects a typical physiological chewing or occlusal contact orientation rather than purely vertical loading. The crown was embedded in a rigid support medium and secured with a mechanical vice assembly to prevent displacement or rotational instability during testing, as shown in Figure 11. Vertical compressive displacement was then applied through the indenter to the designated contact area of the inclined crown.

Loading was carried out under controlled displacement conditions to enable gradual crack initiation and growth within the zirconia crown. AE signals generated during the damage process were continuously recorded and synchronized with the mechanical loading data. These signals were then processed using the same preprocessing pipeline described earlier (Kalman filtering, normalization, and CWT). The resulting scalograms were input into the Stage 1 CNN2D–BiGRU model for component classification, followed by the Stage 2 ResNet-18 with BiLSTM–Attention model for spatial crack localization.

## 4. Diagnostic Performance and Spatiotemporal Analysis

### 4.1. Component Classification Accuracy and Robustness

#### 4.1.1. Training and Validation Loss of the CNN2D–BiGRU Model

Stage 1 evaluates the binary Root–Crown classification task on a test set of 600 signals (300 per class), derived from the 80%/20% sample-level split of the 3000-signal PLB dataset; this larger test set reflects the binary nature of the task and the balanced two-class structure of Stage 1.

**Figure 12:**
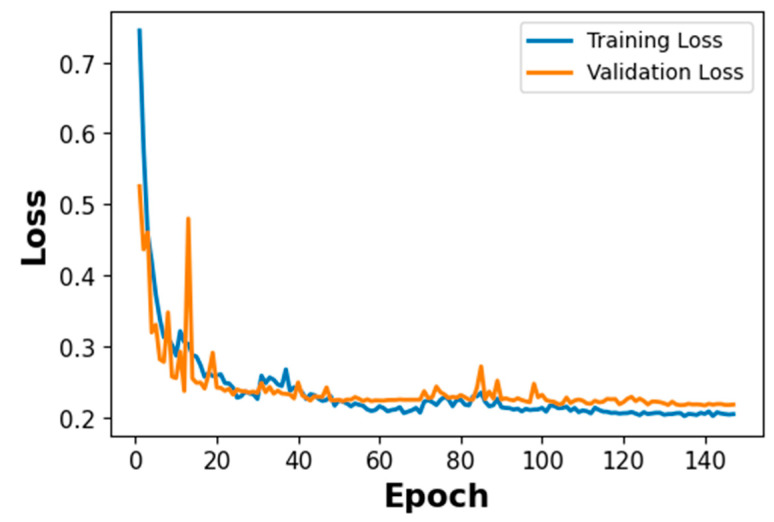
Training and validation loss curves of the CNN2D–BiGRU model.

Figure 12 shows the training dynamics of the CNN2D–BiGRU–Attention model across 150 epochs. Both training loss (blue) and validation loss (orange) decrease steeply from approximately 0.75 in the first 10 epochs, tracking closely throughout with minimal divergence. A brief spike in validation loss is observed around epoch 10–12, after which both curves continue declining and stabilize at approximately epoch 40–50, converging to a stable minimum of approximately 0.20–0.22 for the remainder of training. The close alignment between the two curves from the outset confirms effective regularization and good generalization. Overfitting is controlled through Dropout (*p* = 0.2) after each convolutional block and AdamW with L2 weight decay (1 × 10^−4^), which penalizes large weight magnitudes and prevents the model from fitting noise in the CWT scalogram features.

#### 4.1.2. Confusion Matrix Evaluation of the CNN2D–BiGRU Model

**Figure 13:**
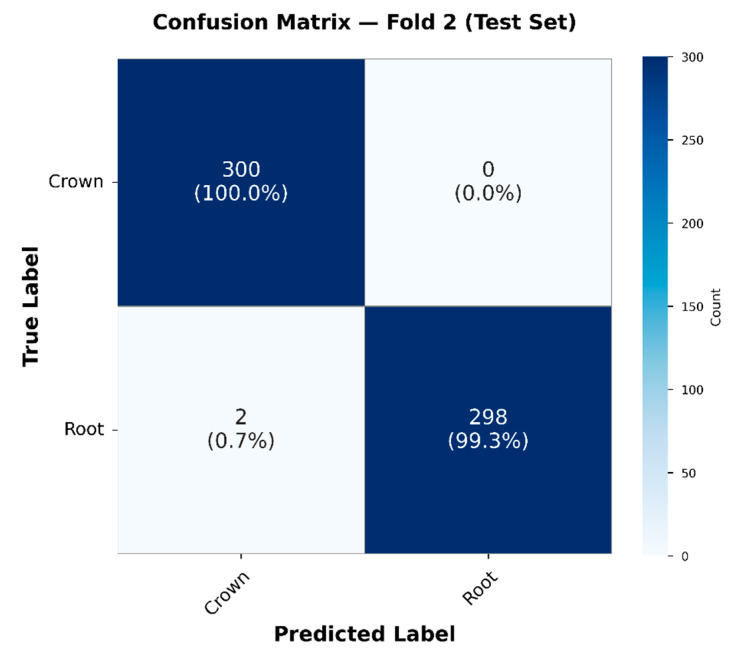
Confusion Matrix of the CNN2D–BiGRU of the CNN2D–BiGRU Model.

To further evaluate the classification reliability and error distribution of the proposed model, a confusion matrix analysis was performed as shown in Figure 13.

Out of 300 signal crown samples, all were correctly classified (100% sensitivity). For the root, 298 out of 300 samples were accurately identified, with 2 misclassified as crown. The overall classification accuracy was 99.67%. Regarding error distribution, the false positive rate for the crown model was very low, with only 0.67% of root samples assigned to the crown class. This indicates strong class separability between the two categories. The confusion matrix shows clear diagonal dominance, further confirming the model’s ability to reliably distinguish between root and crown AE signatures.

These findings are further supported by the quantitative performance metrics summarized in Table 8. Both classes achieved precision and recall values exceeding 0.99, resulting in a macro-averaged F1-score of 0.9967. The nearly identical F1-scores for Crown and Root indicate balanced classification performance without bias toward either class. This high component-level discrimination ensures reliable routing to Stage 2 within the hierarchical diagnostic framework.

#### 4.1.3. Receiver Operating Characteristic (ROC) Curve of the CNN2D–BiGRU Model

To further evaluate the discriminative ability of the Stage1 binary classifier, ROC analysis was conducted on the test set in this study. The ROC curve is a graphical plot that shows the diagnostic performance of a classifier by plotting the True Positive Rate (TPR) against the False Positive Rate (FPR) at various threshold levels.

**Figure 14:**
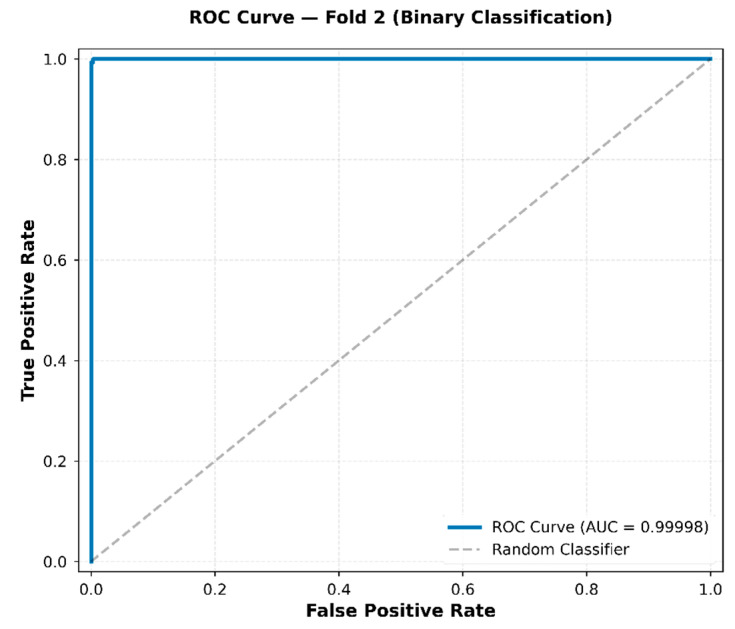
ROC Curve of the CNN2D–BiGRU Model.

As shown in Figure 14, the ROC curve lies almost entirely along the upper-left boundary of the plot, indicating near-perfect separability between the Crown and Root classes. The computed AUC of 0.99998 at Fold 2 indicates strong separability between the Crown and Root classes. The ROC curve lies above the diagonal reference line across all threshold levels.

### 4.2. Precision in Spatial Defect Localization

#### 4.2.1. Training and Validation Loss of ResNet-18 with BiLSTM–Attention Model

**Figure 15:**
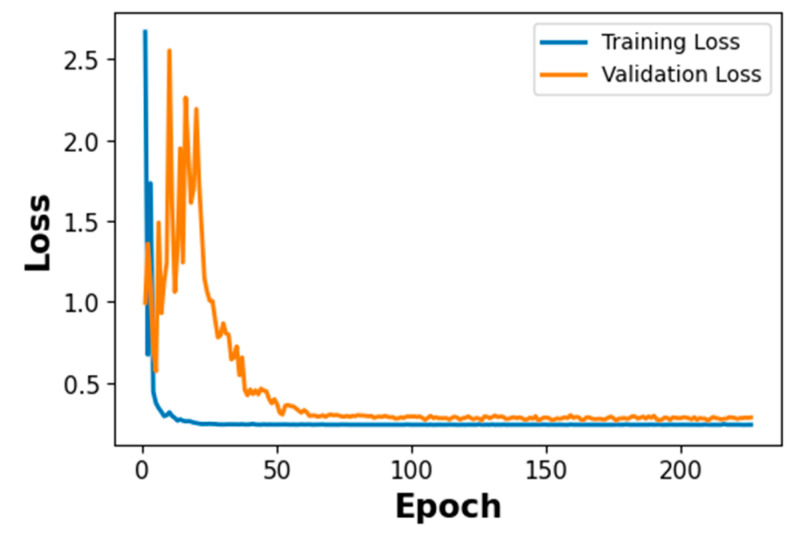
Training and Validation Loss Curves of ResNet-18 with BiLSTM–Attention model for Crown Position Classification.

Stage 2 evaluates the five-class crown position classification task on a test set of 300 signals (60 per class), derived from the 80%/20% sample-level split of the 1500-signal crown PLB dataset. The smaller test set size relative to Stage 1 reflects the five-class structure of Stage 2 and the hierarchical routing design, in which only Crown-classified signals from Stage 1 are forwarded to Stage 2. The training dynamics of the ResNet-18 with BiLSTM–Attention model are shown in Figure 15. The blue line shows the training loss, while the orange line indicates the validation loss over training epochs.

Figure 15 shows the training dynamics of the ResNet-18–BiLSTM–MHA model across 300 epochs. Both training loss (blue) and validation loss (orange) decrease steeply from approximately 0.70 in the first 20 epochs, tracking closely throughout with minimal divergence—reflecting stable learning from the CWT scalogram inputs. Both curves decelerate and stabilize at approximately epoch 50, converging to a minimum of approximately 0.20 for the remainder of training. The close alignment from the outset and the absence of divergence at the plateau confirm effective generalization. Overfitting is controlled through three complementary mechanisms: Spatial Dropout2D (*p* = 0.2) on ResNet feature maps, Layer Normalization within the BiLSTM and attention modules, and AdamW with L2 weight decay (1 × 10^−4^)—the latter penalizing large weight magnitudes across the 27.1 M parameter model. Small residual fluctuations in the validation curve reflect normal batch-level variability and do not indicate instability.

#### 4.2.2. Confusion Matrix Evaluation of ResNet-18 with BiLSTM–Attention Model

**Figure 16:**
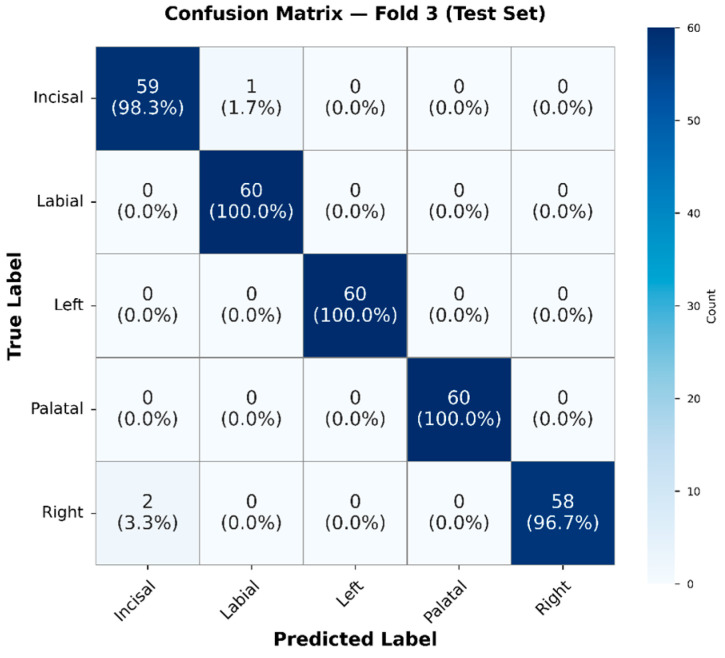
Confusion Matrix of the ResNet-18 with BiLSTM–Attention model for Multi-Class Crown Position Classification.

The proposed model demonstrated high accuracy in predicting the spatial distribution of crown geometry across all five classes: Incisal, Labial, Left, Palatal, and Right. The Labial, Left, and Palatal classes were correctly classified for all 60 samples, achieving 100% accuracy per class. For the Incisal class, 59 out of 60 samples were correctly classified, resulting in a 98.3% accuracy, with one sample misclassified as Labial (1.7%). For the Right class, 58 out of 60 samples were correctly classified, yielding a 96.7% accuracy, with two samples misclassified as Incisal. The resulting confusion matrix is shown in Figure 16.

The confusion matrix is highly diagonal dominant, indicating that the proposed network effectively distinguishes between the spatially varied AE patterns typical for different crown regions. These findings are further supported by the per-class precision, recall, and F1-score values summarized in Table 9.

As summarized in Table 9, the model consistently achieved high precision and recall across all crown regions, with F1-scores above 0.97 for each class. The small performance variations observed in the incisal and right regions match the limited cross-confusion shown in the confusion matrix. This indicates that while spatial patterns are mostly distinctive, certain anatomically adjacent regions may display partially overlapping stress-wave features under inclined occlusal loading.

The overall stability of the per-class metrics further confirms the robustness and spatial discrimination ability of the proposed ResNet-18 with BiLSTM–Attention architecture for precise crown position localization.

#### 4.2.3. Receiver Operating Characteristic (ROC) Curve of ResNet-18 with BiLSTM–Attention Model

**Figure 17:**
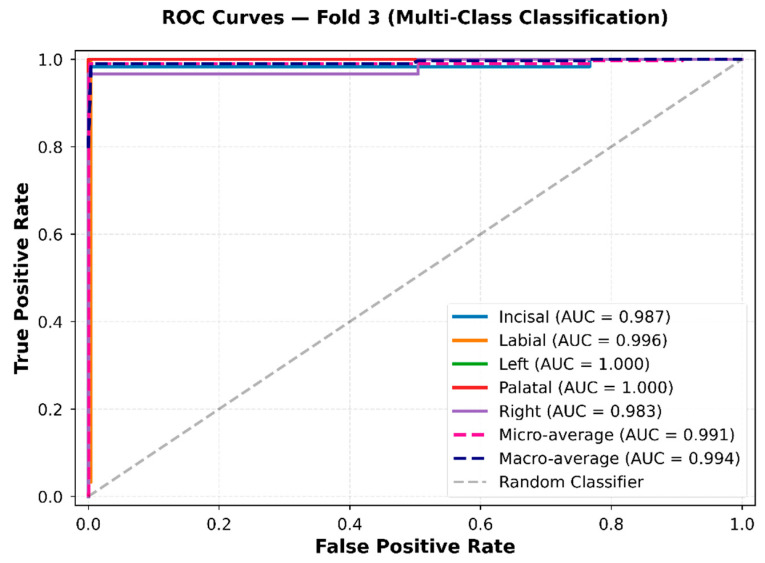
ROC Curves of the ResNet-18 with BiLSTM–Attention for Multi-Class Crown Position Classification.

To further evaluate the discriminative capability of our proposed multi-class classification model, ROC analysis was performed for the five crown regions. The resulting ROC curves are presented in Figure 17.

All classes were well-separated as the ROC curves for each class were close to the top left corner of the ROC space. Corresponding AUC values were 0.987, 0.996, 1.000, 1.000, and 0.983 for incisal, labial, left, palatal, and right classes, respectively, indicating very high classifier performance across all categories. The micro-average AUC was 0.991, while the macro-average AUC was 0.994, demonstrating consistent effectiveness across categories without any class dominating. The ROC curves for all classes are distinctly above the diagonal reference line, demonstrating strong class separability across decision thresholds. The consistently high AUC values align with the confusion matrix and per-class performance metrics.

Overall, the ROC analysis offers further quantitative evidence of stable and consistent multi-class classification performance across the five crown regions under the evaluated mechanical loading conditions.

### 4.3. Five-Fold Stratified Cross-Validation

To assess the robustness and generalizability of the proposed models, five-fold stratified cross-validation was conducted for both Stage 1 and Stage 2. In each fold, the dataset was divided into 80% training and 20% validation partitions while maintaining class balance within each fold. The sample-level separation was maintained throughout all folds, ensuring that no sample contributed signals to both training and validation partitions within any fold. Results are reported as Mean ± SD across all five folds. The five-fold cross-validation results for Stage 1 are summarized in Table 10, whereas the performance of Stage 2 is presented in Table 11.

The five-fold cross-validation results indicate stable performance across different data partitions. For Stage 1, the accuracy standard deviation of ±0.0024 across folds indicates consistent binary classification performance, with all folds exceeding 0.99 accuracy. For Stage 2, the accuracy SD of ±0.0062 reflects slightly greater variability consistent. The highest per-class variability is observed for the Right class (F1 SD = ±0.0108), consistent with the adjacent-based confusion between Incisal and Right. The lowest variability is observed for Palatal (F1 SD = ±0.0042), reflecting the geometrically distinct propagation path of the palatal surface. These results, combined with the sample-level partitioning and the convergent training loss curves in Figure 12 and Figure 15, indicate that the reported performance is consistent across folds and supported by multiple independent lines of evidence against overfitting.

### 4.4. Benchmarking Against Conventional Architectures

A benchmarking analysis was performed against three baseline architectures of increasing representational capacity: CNN2D with a fully connected classifier head (CNN2D + FC), CNN2D with bidirectional LSTM (CNN2D + BiLSTM), and CNN with a Transformer encoder (CNN + Transformer Encoder). All baselines were trained on the same dataset partition and with identical optimizer settings as the proposed model. The quantitative results, including accuracy, F1-score, parameter count, training time per epoch, and accuracy difference relative to the proposed model, are summarized in Table 12.

The benchmarking results demonstrate a consistent improvement in classification accuracy as architectural capability increases. The CNN2D + FC baseline achieved 93.50% accuracy, establishing the performance ceiling of a purely spatial convolutional approach for this task. Incorporating BiLSTM for sequential feature modeling improved accuracy to 96.50%. The CNN + Transformer Encoder achieved 97.50%, demonstrating that self-attention-based sequence modeling provides a stronger baseline than recurrent modeling alone, while still underperforming the proposed approach. The proposed ResNet-18 + BiLSTM + MHA achieved the highest accuracy of 99.00%, maintaining a meaningful advantage over the Transformer-based comparator despite a substantially lower training time per epoch.

### 4.5. Ablation Study: Contribution of Attention Mechanisms

To separate and evaluate the effect of each attention component within the proposed hierarchical framework, ablation assessments were executed for both Stage 1 and Stage 2. In Stage 1, variants were constructed by progressively replacing architectural components—from a fully connected classifier head to unidirectional GRU, then to bidirectional GRU (BiGRU), and finally to the proposed BiGRU with temporal attention—while keeping all other training configurations identical. In Stage 2, a parallel set of variants replaced the sequential and attention modules—from a fully connected head to BiLSTM without attention, then to unidirectional LSTM with Multi-Head Attention (MHA), and finally to the proposed BiLSTM with MHA—with all other hyperparameters fixed. Performance was assessed using accuracy, precision, recall, and F1-score on the held-out test sets: *n* = 600 for Stage 1 (balanced 300/class) and *n* = 300 for Stage 2 (balanced 60/class).

Table 13 and Table 14 confirm that each architectural element provides a discrete, quantifiable enhancement beyond the preceding variant. In Stage 1, replacing the fully connected head with a unidirectional GRU improved the macro F1-score from 0.9383 to 0.9583, demonstrating the value of sequential modeling for AE waveform classification. Upgrading to BiGRU yielded a further gap of 0.0217, confirming that bidirectional processing captures complementary temporal features from both signal directions. Adding temporal attention improved performance from 0.9800 to 0.9967, demonstrating that selective weighting of crack-related burst intervals suppresses background noise and improves discriminative power.

In Stage 2, replacing the fully connected head with BiLSTM improved the macro F1-score from 0.9714 to 0.9797. Adding unidirectional LSTM with MHA yielded a further gap of 0.0050, isolating the contribution of attention-based spatial–frequency refinement independently of bidirectionality. The final upgrade to the proposed BiLSTM + MHA contributed an additional gap of 0.0053, confirming that bidirectional processing further enhances crown region discrimination. 

### 4.6. Inference Efficiency and Deployment Performance

Inference efficiency was evaluated under both single-sample and batch-processing conditions. The results are summarized in Table 15.

Average latency for single sample inference is 12.3 ms, resulting in an average throughput of 81.3 samples/s. In batch mode with 100 samples, the average latency is 847 ms, which translates to an average throughput of 118 samples/s. These results show that the proposed framework keeps low latency and stable throughput, supporting both real-time and batch deployment scenarios.

### 4.7. Generalization to On-Site Dynamic Loading Scenarios

**Figure 18:**
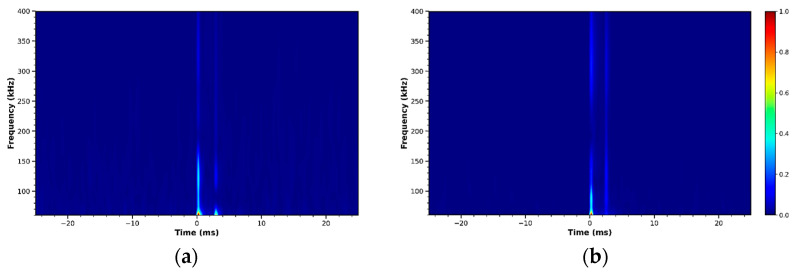
Example scalograms of AE signals acquired during onsite mechanical loading experiments in panels (**a**,**b**).

To assess real-world applicability and evaluate result stability across sample sizes, the proposed hierarchical framework was validated using two onsite mechanical loading datasets: *n* = 30 and *n* = 60 signals, both acquired under the same UTM conditions and loading protocol. The acquired AE signals were processed using the same preprocessing pipeline, including Kalman filtering, normalization, and CWT. Representative CWT scalograms from the onsite mechanical loading experiments are shown in Figure 18.

Despite the increased variability from real loading conditions, the onsite scalograms show time–frequency patterns consistent with those in the PLB-based dataset. These results demonstrate that the extracted time–frequency features remain discriminative and suitable for hierarchical deep learning-based crack classification and localization.

#### 4.7.1. Stage 1 CNN2D–BiGRU Component Classification Onsite Results

**Figure 19:**
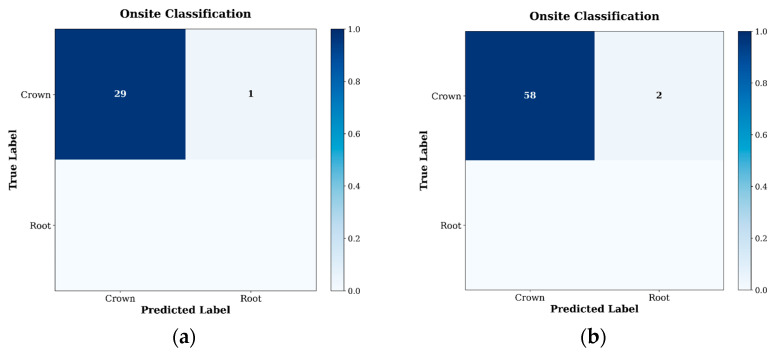
Confusion Matrix of CNN2D–BiGRU Component Classification under Onsite Mechanical Loading Conditions: (**a**) *n* = 30 and (**b**) *n* = 60.

Figure 19a,b present the onsite confusion matrices for Stage 1 at *n* = 30 and *n* = 60, respectively. At *n* = 30, 29 out of 30 samples were correctly classified as Crown, with 1 sample misclassified as Root, yielding an overall onsite accuracy of 96.7%. At *n* = 60, 58 out of 60 samples were correctly classified, with 2 samples misclassified as Root, yielding an identical overall accuracy of 96.7%.

Both confusion matrices show clear diagonal dominance, confirming strong component-level discrimination under realistic loading conditions. The identical accuracy of 96.7% across both sample sizes confirms that the *n* = 30 result is representative and not an artefact of small-sample variability. Misclassifications (1 at *n* = 30; 2 at *n* = 60) are attributable to stochastic signal variability under progressive compressive loading.

#### 4.7.2. Stage 2 ResNet-18 with BiLSTM–Attention Onsite Results

**Figure 20:**
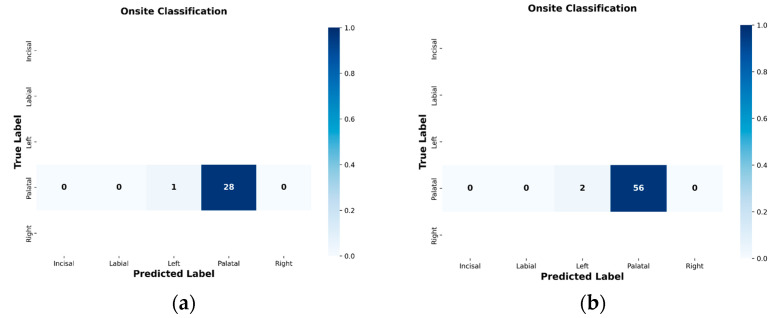
Confusion Matrix of ResNet-18 with BiLSTM–Attention for Crown Position Classification under Onsite Mechanical Loading Conditions: (**a**) *n* = 30 and (**b**) *n* = 60.

Signals identified as Crown in Stage 1 were subsequently forwarded to Stage 2 for spatial crack localization. Figure 20a,b present the onsite confusion matrices for Stage 2 at *n* = 30 and *n* = 60, respectively. At *n* = 30, 29 signals were forwarded; 28 were correctly classified as Palatal, with 1 misclassified as Left, yielding a localization accuracy of 96.6%. At *n* = 60, 58 signals were forwarded; 56 were correctly classified as Palatal, with 2 misclassified as Left, yielding an identical accuracy of 96.6%.

Both confusion matrices remain predominantly diagonal, confirming retained spatial discrimination under real loading conditions. The consistent accuracy of 96.6% at both sample sizes confirms result stability. All misclassifications at both *n* = 30 and *n* = 60 are confined to the Left region, consistent with the anatomical adjacency between the Palatal and Left surfaces and the overlapping wave propagation paths between these proximate crown positions.

The onsite validation results shown in Figure 19 and Figure 20 should be interpreted within the constraints of the experimental configuration. Stage 1 onsite testing involved zirconia crown specimens only and therefore demonstrates Crown-related component recognition rather than full onsite Root-Crown discrimination. Physiologically motivated Class I occlusion-based loading in Stage 2 resulted in predominantly palatal crack initiation. The onsite results thus represent scenario-specific verification of feature transferability to mechanically induced crack events.

In statistical terms, for a binomial proportion of *p* = 0.967 at *n* = 30, the 95% confidence interval is approximately [0.838, 0.999]; at *n* = 60, this narrows to approximately [0.888, 0.999]. The narrower interval at *n* = 60 reflects a more precise estimate of the true classification rate, though both intervals remain wide given the small absolute sample sizes.

### 4.8. Microstructural Corroboration via SEM Analysis

**Figure 21:**
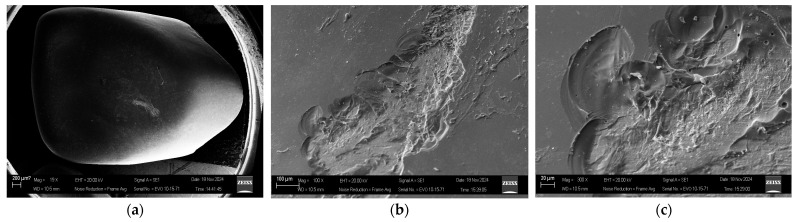
SEM micrographs of the zirconia crown surface after indentation testing from the 60 crown sample subjected to onsite mechanical loading: (**a**) overall surface morphology; (**b**) low-magnification image (100×) showing global deformation features; (**c**) high-magnification image (300×) highlighting crack propagation and chipping around the indentation site.

Test samples after testing with the indentation method were observed using a scanning electron microscope (SEM, EVO MA10, Carl Zeiss, SMT AG, Jena, Germany) to examine the appearance of the indentation and cracks. SEM observation, shown in Figure 21, presents SEM images of the indentation and cracks. The cracks presented a chipping appearance when the indenter was pressed onto the palatal surface simultaneously with the biting area. The surface of the zirconia, a brittle solid, shows an inelastically deformed region immediately beneath the indenter.

### 4.9. Comparative Analysis of Failure Detection Modalities: AE and UTM

**Figure 22:**
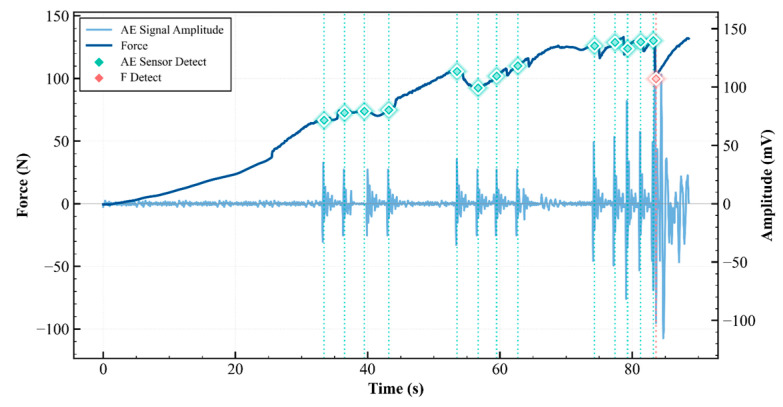
Comparative Force–Time and AE Response during Mechanical Loading, Highlighting Early AE-Based Detection and Late UTM Force-Drop Failure Identification.

The Limitation of Traditional Mechanics (The Force curve): In standard biomechanical testing with UTM, failure is usually defined macroscopically. As shown in Figure 22, the dark blue Force curve indicates that the system registers a “failure” or a significant event only when there is a significant drop in load capacity. The red diamond labeled “F Detect” at approximately t ≈ 84 s. This point represents the moment when the mechanical system physically yields enough to activate a force-drop algorithm. By the time the force drops, the structural integrity of the sample (such as bone, implant, or scaffold) has likely already suffered catastrophic damage. This is a lagging indicator.

The Superior Sensitivity of AE signals acts like a “micro-stethoscope” for the material. It detects the temporary elastic waves caused by the rapid release of energy from local origins within the materials such as micro-cracking, breakage, or fiber debonding before a continuous, macroscopic fracture occurs. In the upper panel (light blue line), bursts of high-amplitude energy can be observed with onset as early t ≈ 33 s. The “AE Sensor Detect” events (teal diamonds) are instances where an AE signal exceeds a certain amplitude threshold. These events recur at approximately 33, 36, 40, 43, 53, 56, 62, 74, 78, and 80–85 s. The period between 80 and 85 s marks the critical failure phase, characterized by a rapid succession of high-amplitude AE bursts. As the applied force reaches its peak, the material undergoes significant structural deterioration, evidenced by frequent and sharp declines in the force curve. Mechanical instability causes intense AE activity, culminating in a catastrophic failure around 83 s (red diamond). The clustering of AE signals indicates the shift from a stable cracking phase to an unstable failure phase.

## 5. Discussion

### 5.1. Early Detection Advantage: Early Detection Advantage Under Laboratory Conditions

A particularly important finding from our research is the time advantage of the AE signal over traditional mechanical testing. The hierarchical deep learning framework, driven by real-time AE monitoring, directly addresses this diagnostic gap by enabling early, non-invasive, early-stage crack detection that traditional methods cannot accomplish. The two-stage classification system achieved an overall accuracy of 99.67% at the component level (Stage 1) and 99.00% at the spatial localization level (Stage 2), with consistent results verified in onsite mechanical loading tests. As shown in Figure 22, the AE system detected the earliest signs of internal material stress around t = 33 s, while UTM registered macroscopic failure—indicated by a force drop—only at about t ≈ 84 s in this sample under these loading conditions [41]. These results collectively show these results indicate that the framework maintains classification performance under controlled mechanical loading conditions; clinical applicability requires further validation under physiological conditions.

If validated clinically, early detection of microcrack activity could in principle inform occlusal adjustment or prosthetic intervention before catastrophic fracture; however, prospective clinical studies are required to establish actionable thresholds and site reconstruction [42].

### 5.2. Physical Interpretation of AE Signal Generation and Propagation in Dental Zirconia

#### 5.2.1. Mode I (Tensile Opening) and AE Generation

Zirconia, as a brittle ceramic characterized by limited plastic deformation capacity, predominantly fails via Mode I (tensile opening) crack propagation driven by tensile stress concentrations at surface flaws or subsurface pores. This fracture mechanism involves abrupt atomic bond rupture, which spontaneously releases stored elastic strain energy in the form of transient stress waves, fundamentally generating AE events [43]. The elastic modulus (E ≈ 210 GPa) and density (ρ ≈ 6.05 g/cm^3^) of sintered Y-TZP zirconia [44] dictate the longitudinal wave velocity (~6000–6500 m/s). Mode I crack nucleation is therefore commonly associated with short-duration, high-frequency-dominated AE bursts, reflecting rapid crack-face opening and elastic energy release, whereas subsequent signal components may arise from wave interactions within the bulk technical ceramics, including partially stabilized zirconia (PSZ) [45]. The AE sensor employed in this study (WM500/1; resonant frequency 500 kHz; operating range 100–1000 kHz) was specifically selected to capture this full frequency range of crack-induced AE in zirconia, consistent with wideband piezoelectric sensor configurations recommended for brittle ceramic monitoring [43].

#### 5.2.2. Mode II/III (Shear Sliding) and Continuous Wavelet Transform (CWT)

Conversely, while less prevalent in typical zirconia applications, Mode II (in-plane shear) and Mode III (out-of-plane tearing) fractures involve frictional sliding and crack-face contact along preferential grain boundaries. These shear-related processes involve frictional sliding and crack-face interaction and are generally characterized by longer-duration AE activity with relatively lower dominant frequency content compared with tensile cracking. To decouple and analyze these overlapping physical phenomena, CWT is employed to map the recorded AE signals into the time–frequency domain [46]. The effectiveness of CWT in isolating distinct damage mechanisms into specific, identifiable frequency bands on the scalogram is well documented in composite materials evaluation.

In the present study, the critical insight motivating the application of CWT within this framework lies in the unobstructed correlation between physics-determined spectral ranges and learnable image characteristics. By mapping mode-dependent acoustic signatures into distinct regions of the scalogram, CWT enables the CNN component of the proposed architecture to implicitly learn fracture-mode-specific features without requiring explicit mode labeling. The high-frequency burst patterns characteristic of Mode I initiation, the mid-frequency oscillations associated with shear propagation, and the low-frequency continuum from structural resonance each occupy spatially distinct regions of the scalogram image, providing a physically meaningful feature space for hierarchical classification. A comprehensive quantitative delineation of individual fracture modalities and the creation of mode-specific acoustic signature repositories were outside the scope of this study and are recognized as significant avenues for subsequent inquiry.

### 5.3. Clinical Implications for Complex Implant-Supported Rehabilitations

Technical complications in implant-supported zirconia fixed dental prostheses account for a significant portion of failures. The economic burden is considerable, not including potential surgical costs if implant integrity is compromised. Additionally, prosthetic failure during the early post-loading phase may jeopardize osseointegration, especially in patients with compromised bone quality. The ability to implement continuous SHM through an AE-based diagnostic platform could fundamentally change the management approach for high-value, high-risk implant rehabilitations. Instead of relying on reactive protocols activated after failure, medical professionals might consider implementing a predictive maintenance strategy, similar to those used effectively in aerospace and civil engineering [47]. For example, integrating miniaturized AE sensors directly into prosthetic frameworks could enable passive, long-term monitoring during normal patient activity, with real-time alerts triggered when cumulative damage surpasses predefined safety thresholds [48].

The integration of smart diagnostic technologies into routine implant maintenance workflows suggests that future research should prioritize non-invasive monitoring systems capable of detecting damage at the microstructural level. The AE-based deep learning framework presented in this study aligns closely with these suggestions. Under laboratory conditions, it demonstrates the ability to detect early microcrack activity with a lead time exceeding 50 s relative to UTM force-drop failure, achieves spatial localization accuracy of 99.00%, and operates with minimal inference latency (12.3 ms per sample), collectively meeting the key performance benchmarks under the evaluated conditions. Furthermore, the hierarchical diagnostic architecture—comprising both component-level (Root vs. Crown) and spatial (5-class crown position) classification—provides the multi-scale diagnostic resolution necessary for clinical decision-making in complex implant cases.

### 5.4. Interpretation of Stage 1 Component Classification Performance

The Stage 1 CNN2D-BiGRU model achieved near-perfect binary classification between Crown and Root components, with an overall accuracy of 99.67%, a macro-average F1-score of 0.9967, and an area under the ROC curve (AUC) of 0.99998. This exceptional performance demonstrates the model’s ability to identify distinctive acoustic signatures associated with different structural configurations, particularly the hollow cylindrical root fixture compared to the complex crown morphology. The clear scalogram patterns seen between these two component types (Figure 2) result from fundamental differences in elastic wave propagation mechanics. The hollow cylindrical root structure displays characteristic guided wave modes with well-defined dispersion relationships, while the crown’s irregular surface topology and variable thickness contribute to mode conversion, scattering, and multipath propagation effects [49]. CWT effectively captured these spectral features dependent on geometry, allowing the CNN to extract hierarchical spatial-frequency information. Adding BiGRU improved classification by better capturing the temporal dynamics of AE signals. Unlike traditional feedforward models, BiGRU analyzes sequential data in both directions, capturing long-range dependencies and transient features related to crack-induced AE [50]. The temporal attention mechanism applied on top of the BiGRU layer dynamically prioritized the most informative time segments, effectively reducing background noise and irrelevant signal components (such as patient speech or equipment vibration).

From a clinical diagnostic standpoint, accurate component-level discrimination is crucial. Crown-level damage usually requires prosthetic intervention (e.g., restoration adjustment, replacement), while root or implant-level damage may necessitate surgical management, including implant removal, guided bone regeneration, and site rehabilitation [51]. Under laboratory conditions, the framework achieved over 99% accuracy for component-level discrimination; comparison with clinical imaging methods requires validation in equivalent clinical conditions. Clear information allows for selecting the most appropriate and precise treatment, enhancing the patient’s benefits.

### 5.5. Interpretation of Stage 2 Spatial Localization Performance

The Stage 2 ResNet-18 with BiLSTM and Multi-Head Attention architecture demonstrated robust five-class spatial localization within the zirconia crown sample, achieving an overall accuracy of 99.00% with per-class F1-scores consistently exceeding 0.97 for all anatomical regions (Incisal, Labial, Left, Palatal, Right). This performance level is significant due to the complex three-dimensional geometry of dental crowns and the inherently anisotropic, inhomogeneous characteristics of stress-wave propagation in curved structures [52]. The minor classification ambiguity observed between Incisal and Right regions (Figure 14) is mechanistically interpretable. Due to the anatomical proximity of the incisal and proximal (right) surfaces, overlapping wavefront arrival times at the sensor may produce similar time–frequency energy distributions, resulting in occasional misclassification. This phenomenon aligns with AE source localization theory, predicting reduced spatial resolution for proximate sources without multi-sensor triangulation [53].

The analysis in Table 10 demonstrates the advantages of architectural complexity. The baseline CNN2D with fully connected layers achieved 93.50% accuracy, which is respectable but insufficient for clinical deployment. Incorporating BiLSTM improved performance to 96.50%, showing the importance of sequential modeling for transient AE signals. The proposed ResNet-18 with BiLSTM and Multi-Head Attention reached 99.00% accuracy, confirming that deep residual feature learning, bidirectional temporal modeling, and attention-based feature refinement contribute synergistically to diagnostic performance [54]. From a clinical perspective, the ability to spatially localize crack initiation sites within the crown structure is diagnostically meaningful. For example, cracks starting at the palatal surface—as confirmed by SEM analysis in this study (Figure 18)—are frequently associated with non-axial loading forces, which are more common in patients with deep overbite, Class II malocclusion, or parafunctional clenching habits [55]. Early detection of palatal surface damage could lead to timely occlusal adjustment (such as selective grinding to reduce interceptive contacts) or the manufacturing of a protective dental occlusal splint before a catastrophic fracture occurs [56]. Similarly, detecting labial surface damage may indicate excessive anterior guidance or traumatic force, necessitating examination of patient-specific risk factors.

### 5.6. Advantages over Conventional Nondestructive Evaluation (NDE) Methods

The main goal of this study was to overcome the diagnostic limitations of current NDE techniques used in dental ceramic structures. As discussed in the introduction, conventional imaging methods face notable constraints. In contrast, the proposed AE-based deep learning system introduces a fundamentally different approach to diagnosis. It operates non-invasively, without the need for ionizing radiation, thereby avoiding safety concerns related to cumulative imaging exposure. The system provides real-time monitoring during laboratory testing and clinical loading conditions, allowing continuous assessment instead of isolated snapshots. It shows high sensitivity to micro-scale events through wideband piezoelectric sensors covering 100–1000 kHz, a range specifically tuned to detect acoustic signals from microcracks in zirconia ceramics [57]. Importantly, the system achieves spatial localization without multi-sensor arrays or complex tomographic algorithms, simplifying equipment and computation needs [53]. Its inference latency of 12.3 ms per sample demonstrates its ability to support near real-time deployment. Moving from episodic checks to continuous SHM marks a paradigm shift similar to transitioning from scheduled aircraft inspections to real-time flight data analysis in aerospace engineering [47], offering great potential to enhance the long-term reliability and safety of high-value implant restorations.

### 5.7. Limitations and Directions for Future Research

Several limitations in this study define clear pathways for future research. The framework was trained on PLB-generated signals [34], which, while ASTM E976-compliant and reproducible, represent a standardized surrogate for actual intraoral fracture events; the reported performance should therefore be interpreted as a laboratory validation rather than a direct prediction of clinical accuracy. The experimental protocol also omitted physiological and environmental factors present in the oral cavity, including saliva, soft-tissue coupling, oral biofilm, thermocycling, and long-term fatigue loading, each of which may affect the physical characteristics of recorded AE signals and the generalizability of the learned features. The acoustic transmission rod employed in this study partially mitigates these influences by conducting stress waves through a direct-contact solid pathway that bypasses the saliva and soft-tissue layers; however, full clinical validation remains necessary. Future work should incorporate hydrated loading with artificial saliva, thermocycling up to 10,000 cycles [58], biofilm simulation, fatigue testing exceeding one million masticatory cycles, and multi-sensor configurations to improve spatial localization. Device miniaturization and the establishment of patient-specific AE thresholds linked to clinical risk criteria represent further prerequisites for translational deployment.

#### 5.7.1. Sensor Placement Sensitivity

A single AE sensor mounted at a fixed labial position introduces path-length-dependent attenuation across the five crown regions; events from palatal and incisal surfaces travel farther and arrive with reduced amplitude. The inter-class confusion between Incisal and Right regions (Section 4.2) is consistent with this effect. Multi-sensor arrays with triangulation capability are identified as a priority for future work.

#### 5.7.2. Geometric and Loading-Induced Constraints in Onsite Validation

Specimen geometry and loading configuration further restrict onsite validation. The axisymmetric cylindrical root analog with apical sensor placement provides similar propagation distances from different lateral positions, thus restricting root-level spatial differentiation primarily to component discrimination. Stage 2 loading of Class I occlusion type in Dentistry preferentially induced initiation of a palatal crack, which limited the diversity of the onsite crack locations. These configurations improve clinical relevance but limit onsite evidence to scenario-specific validation. Future work will address these limitations with different loading directions, multi-contact loading, multi-sensor acquisition, and anatomical diversity of specimens.

#### 5.7.3. Signal Attenuation in Complex Crown Geometry

The irregular crown geometry introduces multipath propagation, mode conversion, and region-dependent attenuation. The model was trained on a single crown geometry; generalizability across different crown morphologies, restoration designs, and zirconia formulations (e.g., 3Y-TZP vs. 5Y-PSZ) has not been assessed and requires future validation.

#### 5.7.4. Clinical Applicability Constraints

This study demonstrates feasibility under controlled laboratory conditions. The intraoral environment introduces additional constraints—including ambient acoustic noise, variable occlusal loading, and adjacent tooth structures—that may generate spurious AE signals. Patient-specific diagnostic thresholds linking AE activity to clinically actionable microcrack severity have not yet been established and require prospective clinical studies.

#### 5.7.5. Real-Time Deployment Challenges

While the achieved inference latency of 12.3 ms supports near-real-time monitoring under laboratory conditions, clinical deployment requires additional system-level developments. These include miniaturization of AE sensor and preamplifier hardware, wireless data transmission, power management, and robust signal conditioning in a noisy clinical environment. Regulatory approval and integration with dental chair instrumentation represent further prerequisites for translational use.

From a signal interpretation perspective, the present study treats the CWT scalogram as a holistic time–frequency representation rather than explicitly decomposing individual fracture mode contributions. Although high-energy burst components observed in the upper spectral bands of the scalogram are consistent with brittle crack initiation processes, and lower-frequency components are commonly associated with shear-related mechanisms and stress-wave reverberation, these associations are interpreted as physics-informed trends rather than definitive mode identifiers. AE spectral characteristics are additionally influenced by wave propagation distance, attenuation, geometric dispersion, and sensor coupling.

Accordingly, a detailed quantitative separation of Mode I tensile cracking, Mode II shear mechanisms, and frictional sliding signatures—and the establishment of mode-specific acoustic signature libraries for zirconia dental structures—remains beyond the scope of the present study. Addressing this limitation through multi-sensor acquisition, controlled multi-mode loading, and microstructure-resolved validation represents an important direction for future research and would further strengthen the physical interpretability of AE-driven deep learning frameworks in dental applications.

## 6. Conclusions

This study introduces a hierarchical deep learning method for early crack detection and spatial localization of cracks in zirconia dental materials using AE signals. The approach integrates signal preprocessing, CWT-based time–frequency representation, and a two-phase deep learning architecture. Under controlled laboratory conditions, the Stage 1 CNN2D–BiGRU model achieved 99.67% accuracy with an AUC of 1.000 for Root–Crown classification. The Stage 2 ResNet-18–BiLSTM–Attention model reached 99.00% accuracy with per-class F1-scores above 0.97 for five crown regions. Comparative benchmarking and ablation analyses confirmed that each architectural component contributes an independent performance and measurable improvement over baseline models, and inference testing indicated low latency (12.3 ms per sample), enabling near real-time monitoring. To evaluate robustness beyond controlled excitation, onsite testing under mechanical load conditions confirmed consistent performance, with 96.7% accuracy for component-level classification and approximately 96.6% accuracy for spatial crack localization. SEM observations provided microstructural confirmation of crack initiation features, supporting the AE-based diagnostic results. The present validation is conducted under controlled laboratory conditions using PLB-generated training signals as a physics-consistent baseline; onsite UTM-induced cracks provide preliminary evidence of generalization. Full clinical translation will require further evaluation under physiological intraoral conditions including hydrated loading, thermocycling, and long-term fatigue testing and expanded multi-sample datasets. Within these defined boundaries, the proposed framework demonstrated the feasibility of combining AE sensing with physics-informed deep learning for intelligent crack monitoring and spatial localization in zirconia dental structures, providing a foundation for digital dentistry.

## Figures and Tables

**Table 1:** Material specifications of SHOFU Disc ZR Lucent.

Parameter	Specification
Zirconia material	SHOFU Disc Lucent
Material type/classification	High-translucent pre-sintered zirconia (5Y-PSZ)
Flexural strength	>1000 MPa
Translucency	High
Disk diameter	~98.5 mm
Shade/Layers	Monolayer or multilayer with gradual shade gradient (incisal ~30% to dentin/cervical ~35%)
Sintering temperature	~1450 °C
Thermal expansion coefficient	~10.2 × 10^−6^ K^−1^ (25–500 °C)
Dimensional verification precision	Digital caliper ± 0.01 mm
Electrical requirements	100–240 V, 50/60 Hz

**Table 2:** Technical specifications of the WM500/1 AE sensor.

Parameter	Specification
Manufacturer	QingCheng AE Institute Co., Ltd., Guangzhou, China
Model	WM500/1
Device type	Passive piezoelectric wideband AE sensor
Operating Frequency Range	0.1–1 MHz
Resonant frequency	500 kHz
Sensitivity peak	>65 dB (ref. V/(m/s))
Operating temperature	−20 to 50 °C
Dimensions (D × H)	4.7 × 4.0 mm
Weight	13 g
Case material	SUS-304 stainless steel
Face material	SUS-304 stainless steel
Protection grade	IP68
Connector type	BNC (integral cable)
Calibration standard	ISO 12714; Metallic Materials—Compression Testing at Room Temperature. International Organization for Standardization (ISO): Geneva, Switzerland, 2015 GB/T 19801; Metallic Materials—Compression Test Method at Room Temperature. Standardization Administration of China: Beijing, China, 2005

**Table 3:** Technical specifications of the Picoscope 4262 data acquisition system.

Parameter	Specification
Manufacturer	Pico Technology, St Neots, UK
Model	Picoscope 4262
Device Type	Digital Oscilloscope (Data Acquisition System)
Number of channels	2 Channels
Resolution	16-bit
Sampling Rate (Maximum)	10 MS/s
Analog bandwidth	5 MHz
Buffer memory	16 MS
Input coupling	AC/DC
Input impedance	1 MΩ‖15 pF
Operating Temperature	5 to 40 °C

**Table 4:** Technical specifications of the Shimadzu AG-100kNX2 universal testing machine.

Parameter	Specification
Model	AG-100kNX2 (AUTOGRAPH AGS-X2 Series)
Testing type	Universal testing machine (tension/compression)
Maximum load capacity	100 kN
Load measurement accuracy	±0.5% of indicated value (ISO 7500-1 Class 0.5)
Load cell precision range	1/500 to 1/1 of rated capacity
Crosshead speed range	0.0001–500 mm/min (stepless)
Crosshead speed accuracy	±0.1%
Maximum return speed	550 mm/min
Software	TRAPEZIUM X-V materials testing software, Shimadzu Corporation: Kyoto, Japan, 2020.
Operating temperature	5–40 °C
Applicable standards	ISO 7500-1; Metallic Materials—Calibration and Verification of Static Uniaxial Testing Machines—Part 1: Tension/Compression Testing Machines—Calibration and Verification of the Force-Measuring System. International Organization for Standardization (ISO): Geneva, Switzerland, 2018 ASTM E4; Standard Practices for Force Verification of Testing Machines. ASTM International: West Conshohocken, PA, USA, 2020 JIS B7721; Testing Machines for Tensile, Compressive and Bending Tests—Verification and Calibration of the Force-Measuring System. Japanese Standards Association (JSA): Tokyo, Japan, 2018

**Table 5:** Hyperparameter Configuration of Hierarchical Models.

Parameter	Stage 1	Stage 2
Task	Classification between Crown and Root	5-Class Crown Position
Training Samples	3000	1500
Train/Test Split	80%/20%	80%/20%
Loss Function	Cross-Entropy	Cross-Entropy
Optimizer	AdamW	AdamW
Learning Rate	1 × $10^{-3}$	5 × $10^{-4}$
Weight Decay	1 × $10^{-4}$	1 × $10^{-4}$
Batch Size	64	64
Max Epochs	500	500
Early Stopping	20 epochs	20 epochs

**Table 6:** Architecture configuration of CNN2D–BiGRU. (Stage 1).

Module	Configuration	Output Size
Input	CWT scalogram	Output Size
Conv Block 1	Conv (3 → 32), 3 × 3, BN + ReLU × 2, MaxPool (2 × 2), Dropout (0.2)	32 × 112 × 112
Conv Block 2	Conv (32 → 64), 3 × 3, BN + ReLU × 2, MaxPool (2 × 2), Dropout (0.2)	64 × 56 × 56
Conv Block 3	Conv (64 → 128), 3 × 3, BN + ReLU × 2, MaxPool (2 × 2), Dropout (0.2)	128 × 28 × 28
Conv Block 4	Conv (128 → 256), 3 × 3, BN + ReLU, MaxPool (2 × 2), Dropout (0.2)	256 × 14 × 14
CNN Output	Feature Map	256 × 14 × 14
Reshape	Spatial-to-Sequence (14 × 14 → 196 steps)	196 × 256
BiGRU	Hidden size = 128, 1 layer, Bidirectional	196 × 256
Attention	Linear (256 → 128), Softmax over 196 steps	256
Classifier	Fully Connected + Softmax (2 classes)	2
Output	Root/Crown	2

**Table 7:** Architecture configuration of ResNet-18 with BiLSTM–Attention. (Stage 2).

Module/Block	Configuration	Output Size
Input	RGB CWT Image	3 × 224 × 224
Preprocessing	Resize + Normalize	3 × 224 × 224
CNN Stem	Conv 7 × 7, s = 2, 64 ch + BN + ReLU + MaxPool 3 × 3, s = 2	64 × 56 × 56
Residual Stage 1	2 × Residual Blocks, 64 ch, s = 1	64 × 56 × 56
Residual Stage 2	2 × Residual Blocks, 128 ch, s = 2 + projection shortcut	128 × 28 × 28
Residual Stage 3	2 × Residual Blocks, 256 ch, s = 2 + projection shortcut	256 × 14 × 14
Residual Stage 4	2 × Residual Blocks, 512 ch, s = 2 + projection shortcut	512 × 7 × 7
Dropout2D	*p* = 0.2	512 × 7 × 7
Spatial-to-Sequence	Reshape 7 × 7 → 49 tokens	49 × 512
BiLSTM	2 layers, bidirectional, hidden size = 512	49 × 1024
Multi-Head Attention	8 heads, hidden dim = 1024	49 × 1024
LayerNorm + Dropout	LayerNorm, *p* = 0.2	49 × 1024
Global Average Pooling	AdaptiveAvgPool1D	1024
Global Max Pooling	AdaptiveMaxPool1D	1024
Feature Fusion	Concatenate (Avg + Max)	2048
FC Layer 1	Linear 2048 → 512 + BN + ReLU + Dropout	512
FC Layer 2	Linear 512 → 256 + BN + ReLU + Dropout	256
FC Layer 3	Linear 256 → 128 + BN + ReLU + Dropout	128
Output Layer	Linear 128 → 5 + Softmax	5

**Table 8:** Classification Performance Metrics of the CNN2D–BiGRU.

Class	Precision	Recall	F1-Score
Crown	0.9934	1.000	0.9967
Root	1.000	0.9933	0.9966
Overall (Macro F1)	-	-	0.9967

**Table 9:** Classification Performance Metrics of CNN2D–BiLSTM.

Class	Precision	Recall	F1-Score
Incisal	0.9672	0.9833	0.9752
Labial	0.9836	1.0000	0.9917
Left	1.0000	1.0000	1.0000
Palatal	1.0000	1.0000	1.0000
Right	1.0000	0.9667	0.9831

**Table 10:** Five-fold stratified cross-validation results—Stage 1: CNN2D–BiGRU component classification Root vs. Crown, *n* = 3000, balanced 1500/class).

Metric	Fold 1	Fold 2	Fold 3	Fold 4	Fold 5	Mean ± SD
Accuracy	0.9917	0.9967	0.9933	0.9950	0.9900	0.9933 ± 0.0024
Precision	0.9917	0.9967	0.9934	0.9951	0.9901	0.9934 ± 0.0024
Recall	0.9917	0.9967	0.9934	0.9950	0.9900	0.9934 ± 0.0024
F1-Score	0.9917	0.9967	0.9934	0.995	0.9900	0.9934 ± 0.0024

**Table 11:** Five-fold stratified cross-validation results—Stage 2: ResNet-18–BiLSTM–MHA crown position classification (5-class, *n* = 1500, balanced 300/class).

Metric	Fold 1	Fold 2	Fold 3	Fold 4	Fold 5	Mean ± SD
Accuracy	0.9867	0.9833	0.9900	0.9800	0.9733	0.9827 ± 0.0062
Precision	0.9871	0.9837	0.9902	0.9806	0.9740	0.9831 ± 0.0059
Recall	0.9867	0.9833	0.9900	0.9800	0.9733	0.9827 ± 0.0062
F1-Score (Incisal)	0.9672	0.9748	0.9752	0.9672	0.9500	0.9669 ± 0.0092
F1-Score (Labial)	0.9917	0.9836	0.9917	0.9917	0.9756	0.9869 ± 0.0067
F1-Score (Left)	1.0000	0.9836	1.0000	0.9836	0.9836	0.9902 ± 0.0083
F1-Score (Palatal)	0.9916	1.0000	1.0000	0.9916	1.0000	0.9966 ± 0.0042
F1-Score (Right)	0.9831	0.9744	0.9831	0.9655	0.9565	0.9725 ± 0.0108

**Table 12:** Performance Comparison of Different Deep Learning Architectures for Crown Position Classification.

Model	Accuracy (%)	F1-Score	Parameters	Training Time per Epoch(s)	Improvement (%)
CNN2D + FC	93.50	0.9340	5.2 M	45	−5.50%
CNN2D + BiLSTM	96.50	0.9645	8.5 M	68	−2.50%
CNN + Transformer Encoder	97.50	0.9747	18.3 M	112	−1.50%
ResNet-18 + BiLSTM + MHA (Proposed)	99.00	0.9900	27.1 M	95	-

**Table 13:** Ablation Study—Stage 1: CNN2D–BiGRU component classification.

Model	Performance Metrics			
	Accuracy	Precision	Recall	F1-Score
CNN2D + FC	0.9383	0.9386	0.9383	0.9383
CNN2D + GRU (unidirectional)	0.9583	0.9585	0.9583	0.9583
CNN2D + BiGRU (without attention)	0.9800	0.9801	0.9800	0.9800
CNN2D + BiGRU + Attention (proposed)	0.9967	0.9967	0.9967	0.9967
Difference (GRU inidirectional to BiGRU)	+0.0217	+0.0216	+0.0217	+0.0217
Difference (Attention gain, BiGRU to proposed)	+0.0167	+0.0166	+0.0167	+0.0167

**Table 14:** Ablation Study—Stage 2: ResNet-18 + BiLSTM + Multi-Head Attention crown position classification.

Model	Performance Metrics			
	Accuracy	Precision	Recall	F1-Score
ResNet-18 + FC	0.9717	0.9715	0.9717	0.9714
ResNet-18 + BiLSTM (without attention)	0.9800	0.9798	0.9800	0.9797
ResNet-18 + LSTM + MHA (unidirectional)	0.9850	0.9848	0.9850	0.9847
ResNet-18 + BiLSTM + MHA (proposed)	0.9900	0.9900	0.9900	0.9900
Difference (BiLSTM to LSTM + MHA unidirectional)	+0.0050	+0.0050	+0.0050	+0.0050
Difference (BiLSTM gain, LSTM unidirectional to proposed)	+0.0050	+0.0052	+0.0050	+0.0053

**Table 15:** Inference Performance Metrics for Operational Deployment.

Metric	Single Sample	Batch (100)
Latency	12.3 ms	847 ms (total for samples)
Throughput	81.3 sample/s	118 sample/s

## Data Availability

The raw data supporting the conclusions of this article will be made available by the authors upon request.
